# Aphrodisiac Principles and other Constituents from the Roots of *Panax quinguefolium* and *Panax ginseng*

**DOI:** 10.1021/acsomega.4c04965

**Published:** 2024-07-18

**Authors:** Kun-Ching Cheng, Hsiu-Hui Chan, Wen-Fei Chiou, Chia-Hung Wu, Yue-Chiun Li, Hao-Ze Li, Ping-Chung Kuo, Tian-Shung Wu

**Affiliations:** † School of Pharmacy, College of Medicine, National Cheng Kung University, Tainan 701, Taiwan; ‡ Taiwan Sugar Research Institute, Tainan 70176, Taiwan; § Department of Chemistry, National Cheng Kung University, Tainan 701, Taiwan; ∥ National Research Institute of Chinese Medicine, Taipei 112, Taiwan; ⊥ School of Post-Baccalaureate Chinese Medicine, China Medical University, Taichung 404, Taiwan

## Abstract

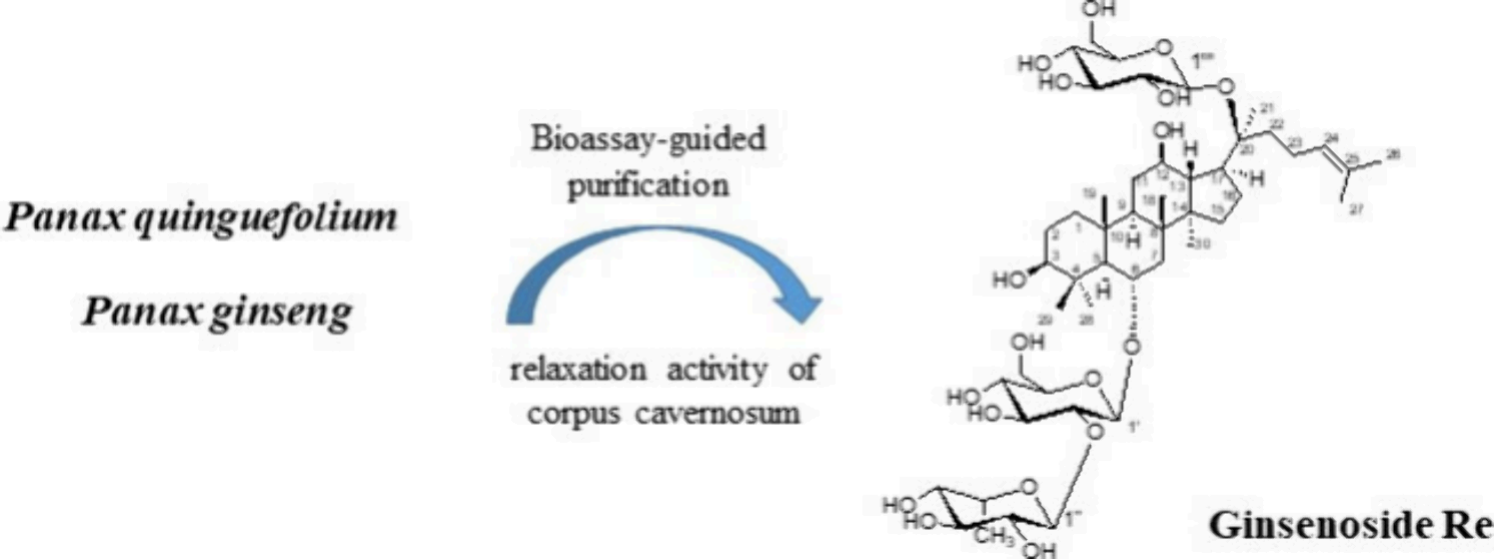

Bioassay-guided fractionation of *P. quinquefolium* and *P. ginseng* root extracts afforded six compounds. Among these, two bioactive compounds ginsenoside Re (**1**) and (20*S*)-ginsenoside Rg2 (**5**) exhibiting significant relaxation in rabbit corpus cavernosum with EC_50_ values of 95.1 and 114.7 μM, respectively. In addition, the phytochemical composition of the water extract of the roots of *P. quinquefolium* was investigated, and thirty-one compounds were characterized, including four undescribed compounds panajaponol B (**18**) and panaxjapynes D-F (**21**–**23**). Moreover, the spectral characteristics and biosynthetic pathway of *Panax* triterpene saponins were discussed according to our results and some previous reports.

*Panax* roots (Araliaceae) have been extensively applied as Chinese herbal medicine or healthy food in East Asian for a long period of time due to their well-known medicinal properties. The pharmacological studies of *Panax* species are extensively reported including the anticancer,^1,2^ immunomodulatory,^3,4^ antiinflammatory,^5^ antiallergic,^6^ neuroprotective,^7^ antihypertensive,^8^ and antidiabetic effects.^9,10^ The phytochemical research previously performed characterize various chemical constituents, and among these triterpenoid saponins further classified into two subgroups based on their aglycons′ skeletons, namely, dammarane- and oleanane-type, is eminent for the broadband bioactivities. The dammarane-type triterpenoid saponins are well-known as ginsenosides, including (20*S*)-protopanaxadiol and (20*S*)-protopanaxatriol. Oleanolic acid type ginsenosides seem to be the typical principles of ginseng species such as *P. ginseng*, *P. japonicus* (Japanese ginseng), and *P. pseudoginseng* subsp. *himalaicus* (Himamayan ginseng), *P. vietnamensis* (Vietnamese ginseng), and *P. zingiberensis* (ginger ginseng).^11^ In our previous publication, the rarely presented polyacetylenes and their inhibitory activities on α-glucosidase were reported from *P. japonicus* C. A. Meyer var. *major*.^12^ For the purpose of exploring new lead compounds from natural sources, this study aimed to investigate the bioactive constituents of the *Panax* roots.

Nowadays most of the research results of male sexual dysfunction were mainly focused on rapid ejaculation (RE) and erectile dysfunction (ED).^13^ The goal for RE therapy was usually to improve patient control over ejaculation timing. Since the walls of the vas deferens, seminal vesicles, ejaculatory ducts, and prostate were lined with smooth muscle cells, the previously published research results indicated that the relaxant agent of smooth muscle could treat RE significantly.^14^ In comparison, the penile arteries and erectile tissue (corpus cavernosum) had to dilate as the erection took place, thereby the blood flow into the penis was increased.^15^ The extents of corpus cavernosal smooth muscle contraction determined the functional states of penile flaccidity (or detumescence) and erection (tumescence). Therefore, exploring some relaxing principles of the corpus cavernosum could be a potential strategy to induce penile erection. In the preliminary bioactivity examination, the water extract of *P. quinquefolium* and methanol extract of *P. ginseng* displayed the significant relaxation of corpus cavernosum with EC_50_ values of 1.67 and 0.26 mg/mL, respectively. Therefore, successive fractionation of the extracts and Diaion HP-20 column chromatography isolation afforded several fractions, and all of the fractions were examined for their relaxation activity of corpus cavernosum. Further purification of the bioactive fractions resulted in several bioactive principles (**1**–**6**). In addition, the phytochemical composition of the water extract of *P. quinquefolium* roots was investigated, and four undescribed compounds panajaponol B (**18**) and panaxjapynes D-F (**21**–**23**) were characterized by the spectroscopic and spectrometric analytical methods. These observations are helpful for the further development of new natural aphrodisiac principles.

## Results and Discussion

**Figure 1:**
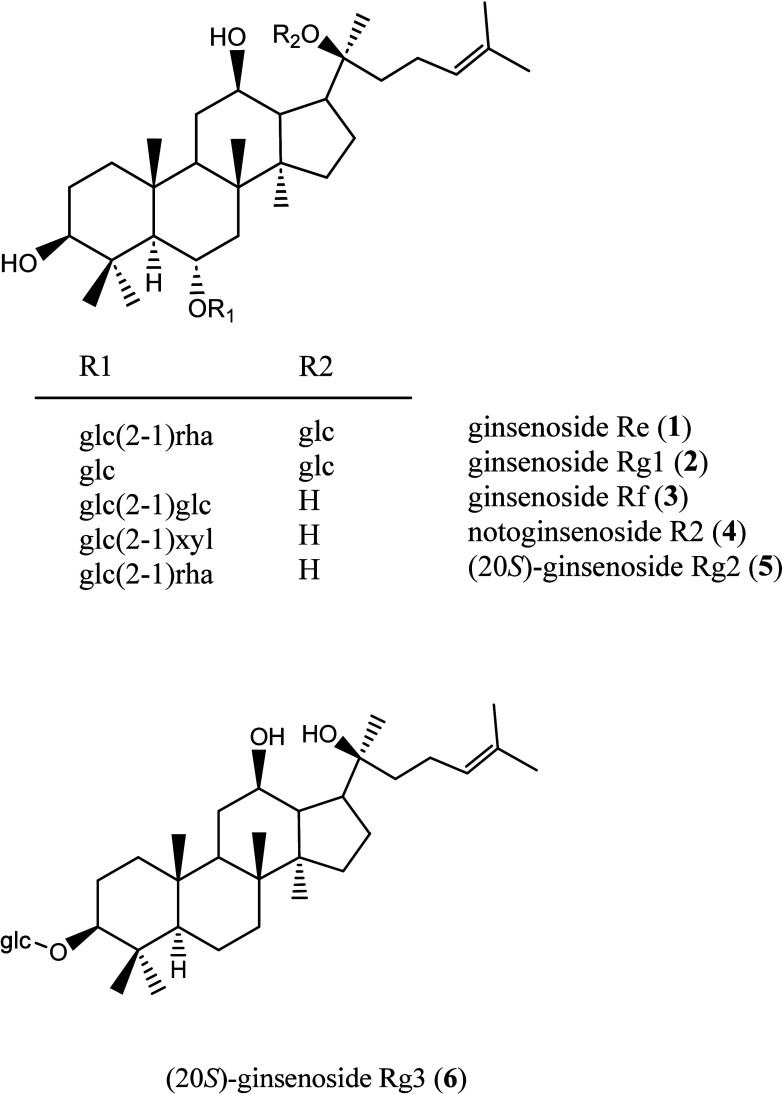
Chemical structures of **1**–**6** isolated from the bioactive fractions.

The roots of *P. quinquefolium* were extracted with water under reflux and concentrated *in vacuo* to obtain a deep brown syrup. The extract was partitioned with ethyl acetate and water to obtain two soluble layers and two batches of precipitates, respectively. The ethyl acetate soluble fraction was subjected to Diaion HP-20 column chromatography to produce four subfractrions. Similarly, the methanol extract of *P. ginseng* was partitioned with ethyl acetate and water to obtain two soluble layers. The successive isolation of this ethyl acetate soluble layer by Diaion HP-20 column chromatography afforded four subfractions. All the resulting layers and subfractions were examined for their relaxation activity of corpus cavernosum of rats, and the precipitates from ethyl acetate layer of *P. quinquefolium* water extracts (PQEP) and the ethyl acetate subfraction of ethyl acetate layer of *P. ginseng* methanol extracts (PGEE) exhibited the most significant relaxation of corpus cavernosum with EC_50_ values of 0.06 and 0.38 mg/mL, respectively (see Supporting Information, Figure S1). Continuous conventional column chromatographic resolution (Figure S2) of these bioactive fractional samples (PQEP and PGEE) totally resulted in six compounds, including ginsenoside Re (**1**),^16^ ginsenoside Rg1 (**2**),^17^ ginsenoside Rf (**3**),^18^ notoginsenoside R2 (**4**),^19^ (20*S*)-ginsenoside Rg2 (**5**),^20^ and (20*S*)-ginsenoside Rg3 (**6**)^21^ (Figure 1). Their chemical structures were identified by comparison of their physical and spectral data with those published in the literature. These purified compounds were examined for their relaxation activity of corpus cavernosum, and among the tested compounds, **1** and **5** displayed the most significant relaxation potential with EC_50_ values of 95.1 and 114.7 μM, respectively (Table 1). It was evident that these bioactive principles were responsible for the aphrodisiac activity of the root extracts of *P. quinquefolium* and *P. ginseng*.

**Table 1:** EC_50_ Values of Relaxation of Corpus Cavernosum of Rats for Purified Compounds

Compound	EC_50_ (μM)
ginsenoside Re (**1**)	95.1
ginsenoside Rg1 (**2**)	349.7
ginsenoside Rf (**3**)	227.7
notoginsenoside R2 (**4**)	155.7
(20*S*)-ginsenoside Rg2 (**5**)	114.7

The above experimental data were further compared with those afforded by molecular docking. Phosphodiesterases (PDE) are enzymes that catalyze the cleavage of the phosphodiester bond of cyclic adenosine monophosphate (cAMP) and cyclic guanidine monophosphate (cGMP). These enzymes control intracellular concentrations of cAMP and cGMP, which play a vital role in mediating the activation of protein kinase to phosphorylate substrates responsible for regulating smooth muscle contraction. cGMP specific phosphodiesterase-5 (PDE5) isoform is expressed in smooth muscle tissue, specifically in the corpus cavernosum.^22^ Many PDE5 inhibitors were clinically approved to be marketed as drugs for treatment of human male erectile dysfunction, including sildenafil (Viagra), vardenafil (Levitra), tadalafil (Cialis), and udenafil (Zydena). Thus, following the relaxation activity of corpus cavernosum examination, a molecular docking study was conducted to determine the binding abilities of purified compounds to PDE5.

**Figure 2:**
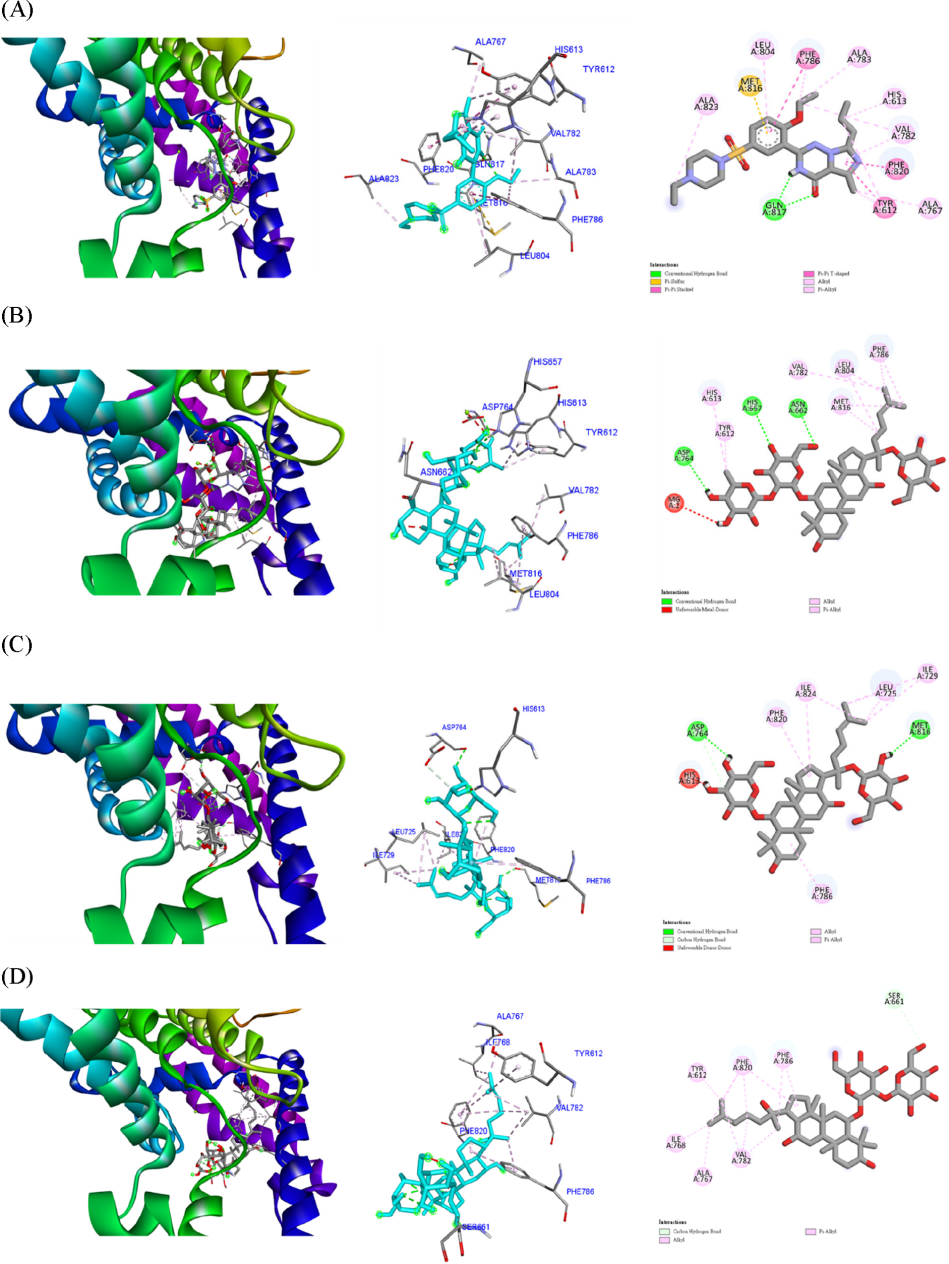

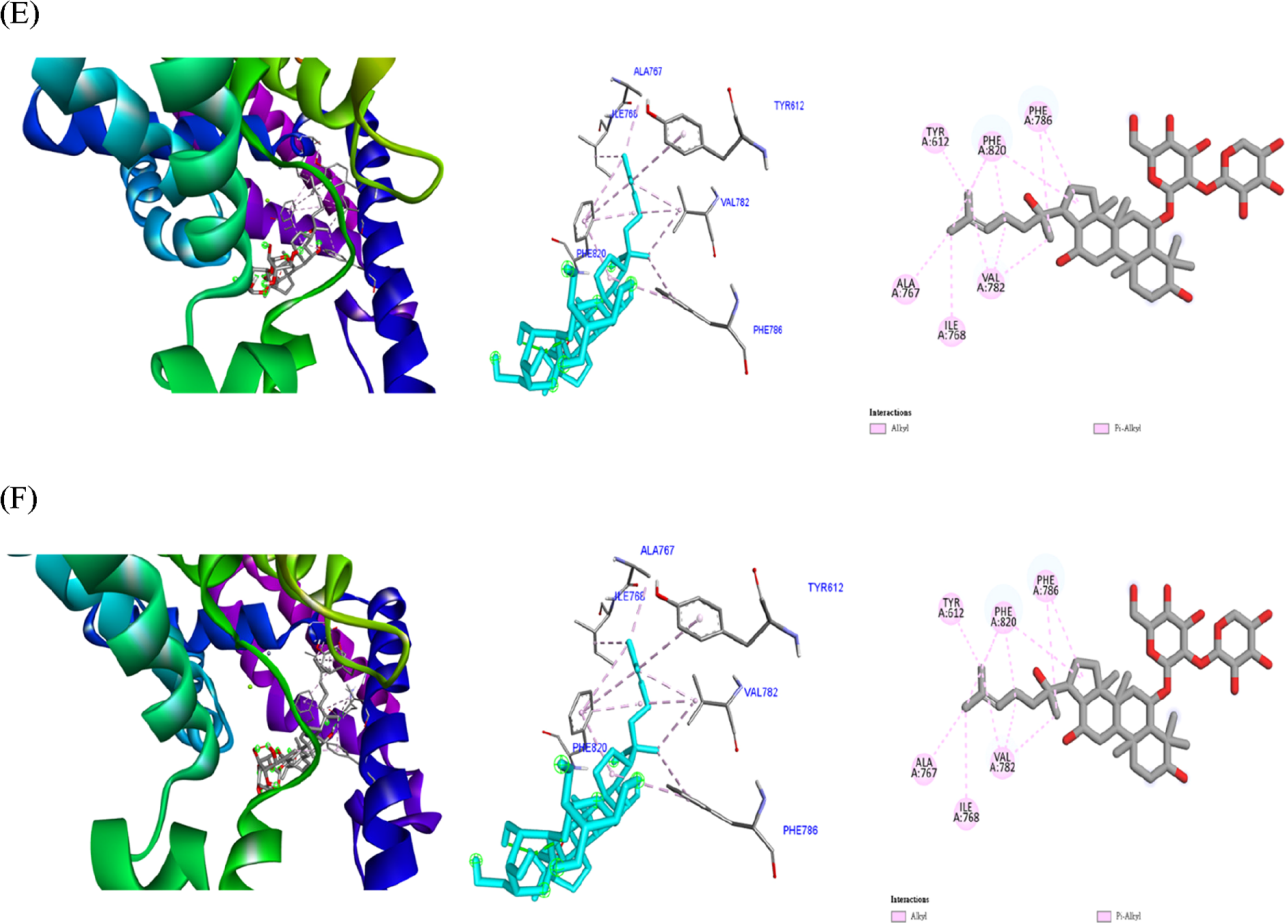
*In silico* modeling of (A) vardenafil (B) **1**, (C) **2**, (D) **3**, (E) **4**, and (F) **5** docking into the PDE5.

In the present molecular docking study, we used ligand vardenafil (a clinically approved PDE5 inhibitor) as a positive control. The calculated binding energy of positive control was −8.9 kcal/mol. Among the molecular docking results (Table 2), all the binding energy values were below −6.0 kcal/mol, which was common value for the selection of potential candidates that are currently accepted in drug design.^23^ The docking results showed that ginsenosides purified from the root extracts of *P. quinquefolium* and *P. ginseng* have similar inhibitory effects on PDE5 as vardenafil. Compounds **1** and **5** revealed the lowest binding energy values of −8.3 and −7.8 kcal/mol, respectively. These *in silico* evaluations were consistent with our aphrodisiac bioactivity examination. By exploring the binding mode interactions of purified compounds and PDE5, the aglycone part of ligands linked proteins with alkyl or pi-alkyl interactions (Figure 2). Compounds **3**, **4**, and **5** showed similar interactions with Phe786, Phe820, Tyr612, Ala767, Ile768, and Val782. Moreover, the residues Asn662, His657, and Asp764 formed hydrogen bond with R1 sugar of compound **1**, probably enhanced the binding affinity between compound **1** and PDE5.

**Table 2:** Binding Energies of Purified Compounds **1-5** And Vardenafil Calculated *In Silico*

Compound	Affinity (kcal/mol)
vardenafil (positive control)	–9.4
ginsenoside Re (**1**)	–8.3
ginsenoside Rg1 (**2**)	–7.1
ginsenoside Rf (**3**)	–7.6
notoginsenoside R2 (**4**)	–7.7
(20*S*)-ginsenoside Rg2 (**5**)	–7.8

*In vivo* and *in silico* studies indicated that compounds **1** and **5** have the most significant potential and lowest binding energy values. Ginsenoside Re (**1**) and (20*S*)-ginsenoside Rg2 (**5**) have two polysaccharides at the C-3 position. These data suggested that two polysaccharides linked to the aglycone at C-3 contribute to aphrodisiac bioactivity and protein binding affinity. In addition, substitution of the C-3 position by α-l-rhamnopyranosyl-(1 → 2)-β-d-glucopyranoside has the advantage in bioactivity and binding affinity. The present results showed a few discrepancies as compare with the literature previously published,^24^ among which ginsenosides Re (**1**), Rg2 (**2**), and Rf (**3**) were not active according to those *in silico* calculations. These differences may originate from the pocket size of the binding protein, and these results should be clarified through more detailed animal experiments.

**Figure 3:**
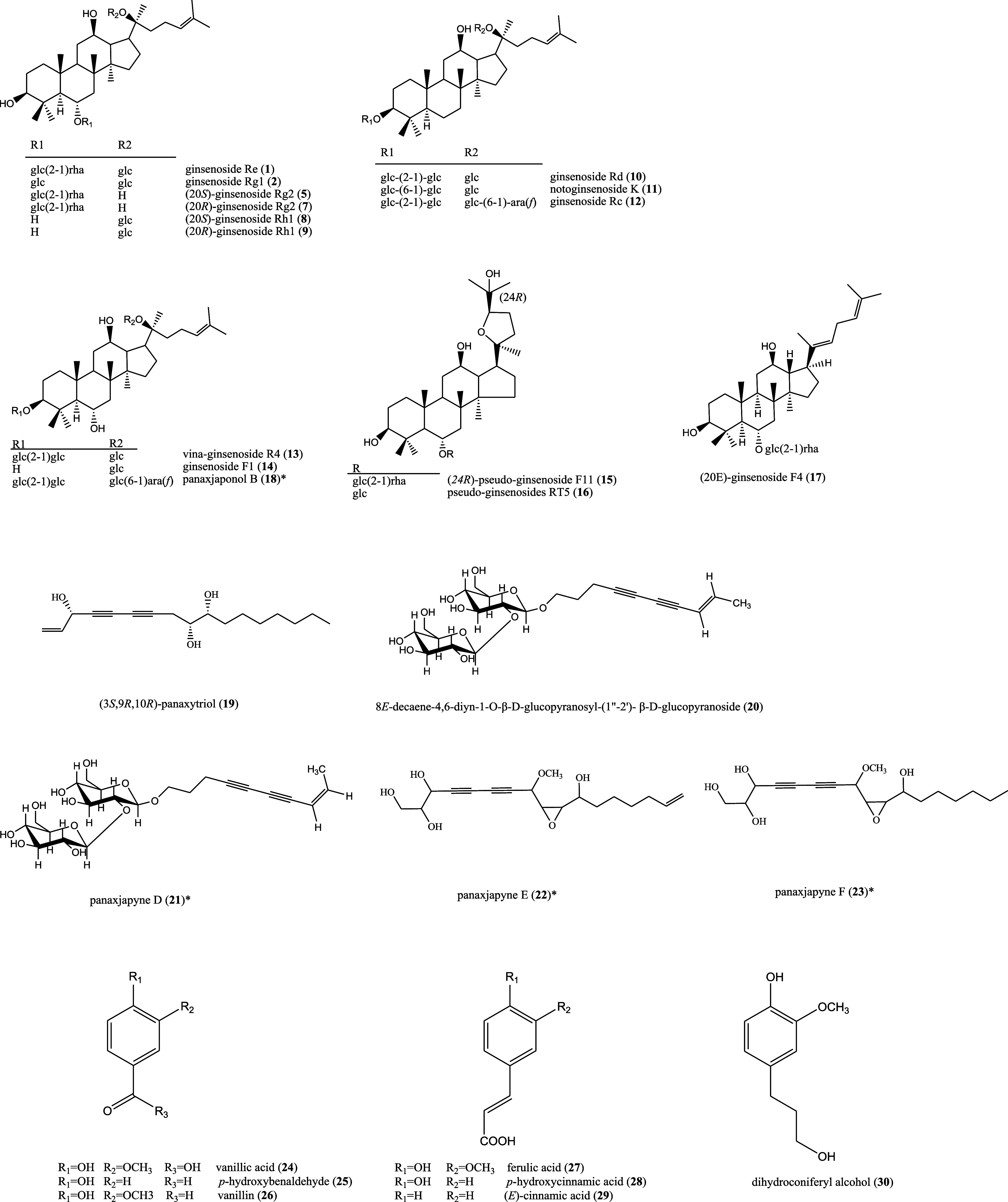

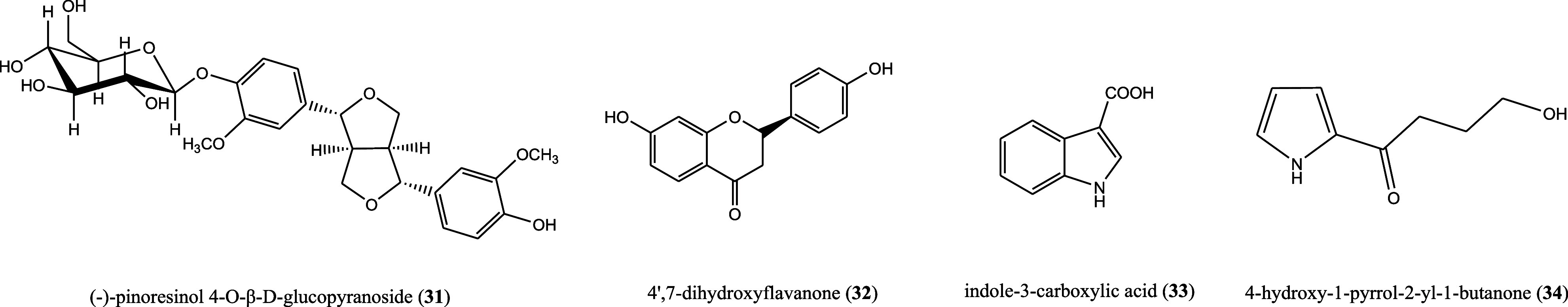
Chemical structures of **1, 2, 5, 7–34** from the ethyl acetate layer of *P. quinquefolium* root extracts.

**Figure 4:**
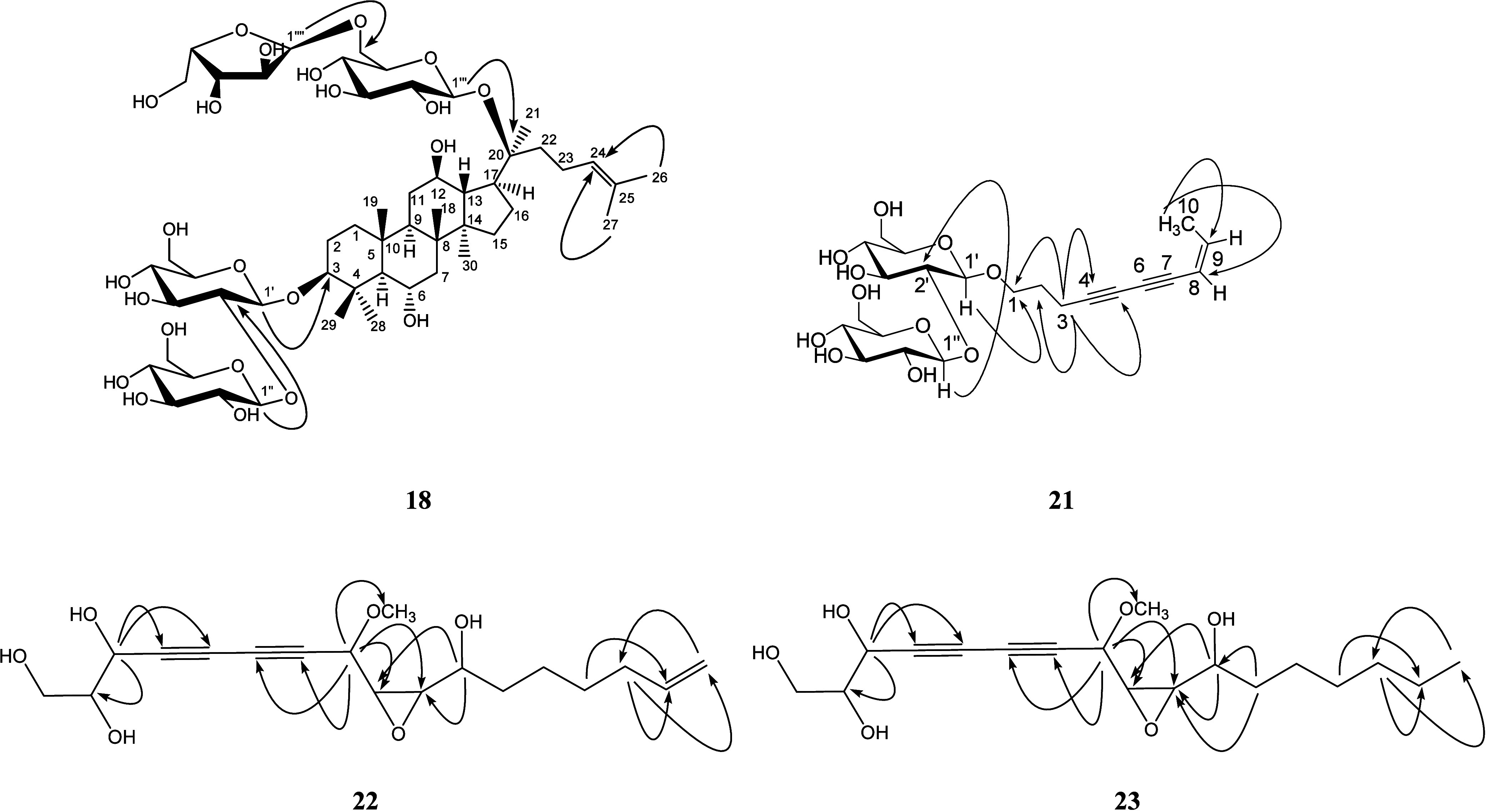
Chemical structures of the previously undescribed compounds **18** and **21**–**23** and significant HMBC correlations (→).

The ethyl acetate layer of *P. quinquefolium* water extracts (PQEE) was also subjected into Diaion HP-20 column chromatography and produced five minor fractions (Figure S3). Further separation resulted in thirty-one compounds (Figure 3); among these, four were reported from the natural sources for the first time, i.e., panajaponol B (**18**) and panaxjapynes D-F (**21**–**23**) (Figure 4). Panajaponol B (**18**) was purified as an optically active colorless amorphous powder (mp 208–210 °C and [α]_D_ + 112.2), and its molecular formula was proposed as C_53_H_90_O_23_ according to the ^1^H- and ^13^C NMR peaks coincided well with the ESI-MS analytical data (*m*/*z* 1118 for [M + Na]^+^) (Figure S4). The IR absorption bands at 3371 (br) and 1670 cm^–1^ were attributed by hydroxyl and carbon–carbon double bond functionalities, respectively (Figure S5). In its ^1^H NMR spectrum (Figure S6), eight singlet methyl groups were observed in the upfield region at δ 1.99, 1.64, 1.61, 1.61, 1.59, 1.09, 0.98, and 0.93. The ^13^C NMR spectrum (Figure S7) also displayed totally fifty-three carbon signals including one oxygenated quaternary carbon at δ 83.4, which were the characteristics of dammarane type triterpenoid saponin. In addition, the detailed comparison and identification of the sugar carbon signals clarified these signals as three β-d-glucoses [δ 105.4 (C-1′), 83.4 (C-2′), 78.1 (C-3′), 71.8 (C-4′), 78.2 (C-5′), 62.9 (C-6′)], [δ 106.0 (C-1″), 77.0 (C-2′′), 78.5 (C-3′′), 71.7 (C-4′′), 78.2 (C-5′′), 62.9 (C-6′′)] and [δ 98.1 (C-1‴), 75.1 (C-2′′′), 79.3 (C-3′′′), 72.1 (C-4′′′), 76.6 (C-5′′′), 68.5 (C-6′′′)], and one α-l-arabinose [δ 110.2 (C-1⁗), 83.4 (C-2′′′′), 78.9 (C-3′′′′), 72.1 (C-4′′′′), 62.7 (C-5′′′′)], respectively. Their absolute configurations were further confirmed according to the HPLC analytical results of acid hydrolysates of **18** by comparing the retention times and optical rotations of the sugars with those of authentic samples as reported previously.^12^ Successive 2D NMR analysis (Figures S8–S11) showed four anomeric protons located at δ 5.65 (1H, d, *J* = 1.2 Hz, H-1⁗), 5.41 (1H, d, *J* = 7.6 Hz, H-1″), 5.12 (1H, d, *J* = 7.6 Hz, H-1‴), and 4.97 (1H, d, *J* = 7.2 Hz, H-1′), as combined with the ^13^C NMR data analysis only the chemical shifts of α-L-arabinofuranose were significantly different from those of floralginsenoside P.^25^ The downfield shift of C-4⁗ suggested the sugar moiety to be α-l-arabinopyranose rather than α-l-arabinofuranose. ^2^*J*,^3^*J*-HMBC correlations could be observed from H-1′ to C-3 (δ 89.5) and from H-1″ to C-2′ (δ 83.4), and the downfield shift of C-2′, indicated the C-3 substitution as β-d-glucopyranosyl-(1′′ → 2′)-β-d-glucopyranoside. Moreover, the HMBC correlation signals of H-1‴ to C-20 (δ 83.4) and H-1⁗ to C-6′′′ (δ 68.5) evidenced the β-d-glucopyranosyl-(1′′ → 2′)-β-d-glucopyranoside group to be located at C-20. In summary, these above spectral data confirmed the structure of **18** as shown and named trivially as panajaponol B according to the previous convention.^12^

Compounds **21**–**23** were purified by high performance liquid chromatography and afforded as an optically active colorless syrup. Their UV spectra displayed absorption maxima at the regions of 280, 266, 252, 239 nm characteristic for the polyacetylene derivatives with conjugated diyne chromophore (Figures S12–S14).^26^ IR analysis also exhibited the typical peaks for the hydroxyl, acetylene, and olefinic functionalities around 3325, 2230, and 1630 cm^–1^ (Figures S15–S17). The molecular formula of **21** was deduced from the HR-ESI-MS analytical data (*m*/*z* 495.1840 for [M + Na]^+^) (Figure S18). In its ^1^H NMR spectrum (Figure S19), a set of *cis* coupled olefinic protons at δ 6.13 (1H, dq, *J* = 11.0, 7.0 Hz, H-9) and 5.56 (1H, dq, *J* = 11.0, 1.5 Hz, H-8), and one methyl group at δ 1.88 (1H, dd, *J* = 7.0, 1.5 Hz, H-10) were recorded. The ^13^C NMR analysis (Figure S20) showed twenty-two carbon signals, identified with the assistance of HMQC (Figure S21) to be one set of conjugated diyne (δ 83.8, 78.1, 71.1, 64.3), one oxygenated methylene (δ 67.9), two methylenes (δ 28.3, 15.5), one olefinic group (δ 141.5, 108.6), one methyl (δ 14.9), and two sets of sugar carbon signals. These sugar carbon signals were resolved as two β-d-glucoses (δ 101.6, 81.6, 76.3, 70.0, 76.4, 61.3; and δ 103.8, 74.7, 76.8, 69.9, 76.5, 61.2) according to the chemical shift and coupling constants of anomeric protons [δ 4.65 (1H, d, *J* = 8.5 Hz, H-1′) and 4.41 (1H, d, *J* = 7.0 Hz, H-1″). In its COSY spectrum (Figure S22), the olefinic proton H-9 (δ 6.13) showed a correlation with CH_3_-10 (δ 1.88). HMBC analytical data (Figure S23) also displayed the ^2^*J*,^3^*J*-correlations from H-10 to C-8 (δ 108.6)/C-9 (δ 141.5), from H-8 (δ 5.56)/H-9 (δ 6.13) to C-6 (δ 78.1), and from H-3 (δ 2.52) to C-1 (δ 67.9)/C-2 (δ 28.3)/C-4 (δ 64.3). All these 2D results constructed the basic skeleton of **21** as the same with that of deca-8-ene-4,6-diyne derivative (**20**), however, the NOESY analysis of **21** (Figure S24) showed the opposite geometry at C-8 double bond since there were NOE cross-peak observed between H-8 and H-9. Moreover, the HMBC correlations from H-1′ (δ 4.65) to C-1 (δ 67.9), and from H-1″ (δ 4.41) to C-2′ (δ 81.6) determined the connectivity of the sugar moiety. Consequently, the complete chemical structure of **21** was unambiguously established as (8*Z*)-deca-8-ene-4,6-diyn-1-β-d-glucopyranosyl-(1″-2′)-β-d-glucopyranoside, and named trivially as panaxjapyne D according to the previous convention.^27^

The molecular formula of panaxjapyne E (**22**) was proposed as C_18_H_26_O_6_ based on the signals presented at its ^1^H- and ^13^C NMR spectra which matched the ESI-MS spectrometric data (*m*/*z* 361 for [M + Na]^+^) (Figure S25). In the ^1^H NMR spectrum of **22** (Figure S26), a set of terminal alkene signals at δ 5.82 (1H, ddq, *J* = 16.4, 10.0, 7.2 Hz, H-16), 5.02 (1H, dd, *J* = 16.4, 2.0 Hz, H-17a), and 4.93 (1H, dd, *J* = 10.0, 2.0 Hz, H-17b) were observed. Its ^13^C NMR spectrum (Figure S27) exhibited 18 carbon signals, which were identified with the assistance of HMQC (Figure S28) as conjugated diynes [δ 77.9 (C-4), 75.9 (C-7), 71.7 (C-6), 70.2 (C-5)], one acetylene group [δ 138.8 (C-16), 114.5 (C-17)], six oxygenated methines [δ 74.3 (C-2), 70.5 (C-8), 69.6 (C-11), 63.9 (C-3), 59.3 (C-10), 56.9 (C-9)], one oxygenated methylene [δ 73.4 (C-1)], one methoxy group (δ 56.9), and four methylenes [δ 34.6 (C-12), 33.7 (C-15), 28.9 (C-13), 24.6 (C-14)], respectively. In its COSY spectrum, the correlations among H-8 (δ 4.16, d, *J* = 2.8 Hz), H-9 (δ 3.25, dd, *J* = 7.2, 4.0 Hz), H-10 (δ 2.96, dd, *J* = 7.2, 4.0 Hz), H-11(δ 3.52, m), and H-12 (δ 1.76, m) could be observed to suggest the skeleton of C-8 to C-12. The COSY correlations (Figure S29) of H-12/H-13/H-14/H-15/H-16/H-17 and H-1/H-2/H-3 also supported the presences of the side chain substitutions including one 1-hydroxy-hept-6-en and 1,2,3-trihydroxypropyl fragments. HMBC analytical data (Figure S30) showed the ^2^*J*,^3^*J*-correlations from H-3 (δ 4.52, d, *J* = 2.8 Hz) to C-2/C-4/C-5 and from H-8 to C-6/C-7/C-9/C-10/OCH_3_ indicated that the conjugated diynes were located at C-4, −5, −6, and −7, and the methoxy group was substituted at C-8. Analysis of the molecular formula and hydrogen deficiency index of **22**, and observation of the upfield chemical shifts of C-9 and C-10 suggested C-9 and C-10 to form an epoxide ring. All of the other COSY/HMQC/HMBC analytical data supported the determination of complete planar structures of **22** as shown. However, due to the difficulties for the hydrolysis of key fragments and no reference groups for the CD examinations, even the NOESY was recorded (Figure S31), and the absolute configurations of the chirality centers were not established and remained unknown.

Compound **23** displayed very similar UV absorption maxima, IR characteristic absorption peaks, and typical ^1^H- and ^13^C NMR signals (Figures S32 and S33) as those of **22**. The most significant variation of its spectral data should be the disappearance of the terminal C–C double bond, which was replaced by a saturated carbon chain indicative of the methyl triplet [δ 0.88 (1H, *J* = 6.4 Hz, H-17)], and this was also supported by its ESI-MS analytical data (*m*/*z* 363 for [M + Na]^+^) (Figure S34). The 2D NMR analyses (Figures S35 and S38), including the COSY and HMBC spectra, both suggested that the basic structure of **23** was the same as that of **22**, and only the terminal olefinic group was saturated in **23**. Conclusively, **23** was determined as shown and named trivially as panaxjapyne F; similarly, the absolute configurations remained undeteremined.

In addition, (20*R*)-ginsenoside Rg2 (**7**),^20^ (20*S*)-ginsenoside Rh1 (**8**),^28^ (20*R*)-ginsenoside Rh1 (**9**),^28^ ginsenoside Rd (**10**),^29^ notoginsenoside K (**11**),^29^ ginsenoside Rc (**12**),^30^ vina-ginsenoside R4 (**13**),^31^ ginsenoside F1 (**14**),^17^ (20*R*)-*pseudo*-ginsenoside F11 (**15**),^32^*pseudo*-ginsenoside RT5 (**16**),^33^ (*20E*)-ginsenoside F4 (**17**),^34^ (3*S*,9*R*,10*R*)-panaxytriol (**19**),^35^ 8*E*-decaene-4,6-diyn-1-*O*-β-d-glucopyranosyl-(1″-2′)-β-d-glucopyranoside (**20**),^31^ vanillic acid (**24**),^36^*p*-hydroxybenaldehyde (**25**),^37^ vanillin (**26**),^38^ ferulic acid (**27**),^39^*p*-hydroxycinnamic acid (**28**),^40^ (*E*)-cinnamic acid (**29**),^41^ dihydroconiferyl alcohol (**30**),^41^ (−)-pinoresinol 4-*O*-β-d-glucopyranoside (**31**),^42^ 4′,7-dihydroxyflavanone (**32**),^43^ indole-3-carbxylic acid (**33**),^44^ and 4-hydroxy-1-pyrrol-2-yl-1-butanone (**34**),^45^ were also identified by comparison of their physical and spectral data with those reported (Figure 3).

According to the present research results, we can clarify the spectral characteristics of these purified triterpenoid saponins. These natural ginsenoside derivatives could be classified into three basic skeletons, i.e. dammarane, ocotillol, and oleanolic acid (Figure S39). The first dammarane type compounds exhibited 30 carbon signals including eight methyls and one set of double bonds resonated at the region of δ 125.0–132.0. Two common subtypes of dammarane basic skeleton were reported, including the protopanaxtriol and protopanaxdiol. For the protopanaxtriol subtype, the C-3, -6, and -20 were hydroxylated and even more glycosylated, but usually only one of C-3 and C-6 would be linked with a sugar moiety. In comparison, in the protopanaxdiol subtype usually only C-3 and -20 were hydroxylated or glycosylated. These two subtypes could be differentiated from the chemical shift of CH_3_-28 and C-5, among which the downfield shift of CH_3_-28 (∼δ 2.00) and C-5 (>δ 60.0) were the indication of protopanaxtriol derivatives. The chemical shift of glycosylated C-3 was close to δ 89.0 compared to the unglycosylated carbon at δ 78.0. In comparison, the chemical shift of glycosylated C-20 was close to δ 83.0 compared to that of the unglycosylated carbon at δ 73.0. Another observed significant signal is one quaternary carbon located at δ 70.0–72.0 representative of C-20 of the dammarane type triterpenoid. The stereochemistry at C-20 could be trivially deduced by the chemical shift of C-22, in which C-20 was assigned as *S* if C-22 was located around δ 35.0–36.0, whereas C-20 was assigned as *R* if C-22 was resonated at the region of δ 42.0–44.0. The second ocotillol type triterpenoids also contained 30 carbon signals including eight methyls; however, there were not any olefinic carbons observed. Usually C-3, C-12, and C-25 were hydroxylated, and sometimes C-6 was also hydroxylated and glycosylated. The absolute configuration at C-24 could be established as *S* if C-24 was located at δ 85.0. In contrast, C-24 would be assigned as *R* while C-24 was downfield shifted to δ 88.0. The third skeleton of these natural triterpenoid saponins belongs to the oleanolic acid basic structure. This type of ginsenoside derivatives exhibited 30 carbon signals including seven methyl groups which were one fewer than those of another two types, and one set of double bond resonated at the region of δ 123.0–144.5 characteristic for the C-12/C-13 olefinic functionality. Among most of the examples, only C-3 was hydroxylated or glycosylated. The C-28 was usually oxidized to a carboxylic acid group and further glycosylated in some cases, resulting in the upfield shift of C-28.

The commonly acceptable biosynthetic mechanism of triterpenoid was well studied in the previous report (Figure S40).^46^ The squalene oxide was arranged in a *chair-boat-chair-boat* conformation and catalyzed by cycloartenol synthase to result in most plant sterols. In contrast, another *chair–chair–chair–boat* conformation of squalene oxide would afford dammarane- and oleanane-type triterpene saponins. The dammarenediol synthase would produce the dammarenyl cation, and further hydration at C-20 usually resulted in the 20*S*-dammarenediols while a few 20*R*- epimer could be observed (Figure S41).^47^ The successive oxidation at C-12 and C-6 yielded protopanaxdiol and protopanaxtriol, and further glycosylation produced the ginsenoside derivatives. The ocotillol type triterpenoids were biosynthesized through the protopanaxtriol intermediate, which was under C-24/C-25 epoxidation and then ring closure of 20*S*–OH to form a tetrahydrofuran 5-membered ring (Figure S42). The oleanane-type triterpene β-amyrin was resulted from the catalysis of β-amyrin synthase, and the oxidation of CH_3_-28 resulted in the commonly observed oleanolic acid which was further glycosylation to produce the oleanolic acid type ginsenoside derivatives (Figure S43).

## Experimental Section

### General Experimental Procedures

Optical rotations of purified compounds were measured by using a Jasco P-2000 digital polarimeter. The ultraviolet (UV) spectra were obtained by a Hitachi U-2001 UV/vis spectrometer. The infrared (IR) spectra were examined with a Jasco FT/IR-4000 FTIR spectrometer. ^1^H (400 MHz), ^13^C (100 MHz), and 2D NMR spectra were recorded on a Bruker Avance III 400 NMR spectrometer using pyridine-*d*_5_, CD_3_OD, or CDCl_3_ as the solvents. Chemical shifts are shown in δ values (ppm) with tetramethylsilane as an internal standard. ESI and HR-ESI mass spectra were measured on a Bruker APEX II mass spectrometer (operated in positive-ion mode). Reversed-phase column chromatography (CC) was accomplished with Diaion HP-20 and Sephadex LH-20 columns. Silica gel column chromatography (SiO_2_ CC) was carried out using a Kieselgel 60 (70–230 and 230–400 mesh, Merck). Thin-layer chromatography (TLC) was executed on precoated Kieselgel 60 F_254_ plates (Merck), with compounds visualized by UV light or spraying with anisaldehyde in 10% (v/v) H_2_SO_4_ followed by charring at 110 °C for 10 min.

### Plant Materials

The extracts of the roots of *P. quinquefolium* and *P. ginseng* were provided by Chuang Song Zong Pharmaceutical Co., Ltd. in Kaohsiung, Taiwan, in 2006. A voucher specimen (TSWu 2006-CSZ-001 and 002) were deposited in Chuang Song Zong Pharmaceutical Co., Ltd. and School of Pharmacy, College of Medicine, National Cheng Kung University, Tainan, Taiwan.

### Extraction and Isolation

The water extracts of the roots of *P. quinquefolium* (PQ, 850 kg) were chromatographed on Diaion HP-20 gel eluted by a water step gradient with ethanol to produce the supernatant (PQEE) and precipitate (PQEP) fractions. A part of the precipitate fraction (PQEP, 46 mg) was isolated by high performance liquid chromatography (HPLC) eluted with water and acetonitrile (15:85) to yield **1** (3.2 mg) and **5** (2.0 mg). The methanol extracts of the roots of *P. ginseng* (PG, 630 g) were partitioned between ethyl acetate and water to obtain the corresponding layers. The ethyl acetate layer (PGEE, 85 g) was eluted on Diaion HP-20 gel by ethyl acetate to afford the eluents, which was further chromatographed on silica gel eluted by ethyl acetate step gradient with methanol to produce nine fractions. The fifth and sixth fractions were isolated by SiO_2_ CC eluted by ethyl acetate step gradient with methanol (19:1 to 9:1), and further purification of the minor fractions by HPLC (water: acetonitrile = 15:85) to yield **2** (59.0 mg), **3** (33.5 mg), and **4** (17.2 mg), respectively. Fractions 7 and 9 were chromatographed on Sephadex LH-20 eluted by a water step gradient with methanol, and the resulting minor fractions were further purified to afford **3** (4.0 mg), **4** (12.1 mg), **5** (10.2 mg), and **6** (31.8 mg), respectively. Compounds **1**–**6** purified from these bioactive fractions were subjected to aphrodisiac bioactivity examination.

The supernatant fraction of *P. quinquefolium* water extracts (PQEE, 213 g) mentioned above was subjected to Diaion HP-20 column chromatography and produced five minor fractions (Fr. 1–5). Fr. Three was isolated by SiO_2_ CC eluted with ethyl acetate and methanol (3:1) to afford five subfractions (Fr. 3.1–3.5). Fr. 3.1 was chromatographed on a column of silica gel eluted with chloroform and methanol (3:1), and further purified by TLC to yield **24** (9.3 mg), **25** (2.3 mg), **26** (1.0 mg), **27** (12.7 mg), **28** (5.3 mg), and **30** (1.0 mg). Fr. 3.2 was also purified by SiO_2_ CC eluted with chloroform and methanol (5:1), and further purified by TLC and HPLC to afford **1** (1.3 g), **2** (2.1 g), **20** (4.2 mg), **21** (3.2 mg), and **31** (1.9 mg). Recrystallization of fraction 3.3 produced a significant amount of needle crystals **1** (11.3 g). Fr. 3.4 was purified by SiO_2_ CC eluted with chloroform and methanol (4:1), and further purified by TLC to yield **1** (570.4 mg), **2** (164.7 mg), **12** (2.0 mg), **13** (2.7 mg), and **18** (7.5 mg). Fr. Four was chromatographed on a column of silica gel and eluted successively with a step gradient of ethyl acetate step and methanol (3:1, 2:1, 1:1) mixture to produce six subfractions (Fr. 4.1–4.6). Fr. 4.1 was purified by SiO_2_ CC eluted with chloroform and methanol (15:1), and further purified by TLC to afford **22** (10.2 mg), **23** (17.5 mg), **27** (3.0 mg), **29** (2.1 mg), and **32** (2.0 mg). Fr. 4.2 was purified by SiO_2_ CC eluted with chloroform and methanol (5:1), and further purified by TLC or recrystallization of the resulting minor fractions to yield **2** (35.2 mg), **5** (5.2 mg), **7** (18.9 mg), **8** (126.5 mg), **9** (39.6 mg), **14** (8.1 mg), **15** (1.3 g), **16** (7.8 mg), **17** (3.7 mg), and **19** (2.5 mg). Fr. 4.3 was also purified by SiO_2_ CC eluted with chloroform and methanol (5:1), and further purified by TLC or recrystallization of the resulting minor fractions to produce **5** (20.5 mg), **7** (22.6 mg), and **15** (6.2 g). Frs. 4.4 and 4.5 were combined and subjected to SiO_2_ CC eluted with chloroform and methanol (3:1), and the resulting minor fractions were further purified by recrystallization or TLC to yield **1** (422.8 mg), **5** (2.1 mg), **7** (1.6 mg), **10** (93.5 mg), **11** (17.5 mg), and **15** (1.2 g). The fifth fraction (Fr. 5) was also purified by SiO_2_ CC, however, there were no significant compounds identified.

Panajaponol B (**18**)**:** colorless powder; mp 208–210 °C; [α]_*D*_^25^ + 112 (*c* 0.1, MeOH); IR (neat) ν_max_ 3371, 2940, 2886, 1670, 1636, 1072, 1038 cm^–1^; ^1^H NMR (400 MHz, pyridine-*d*_5_) δ (ppm) 5.65 (1H, d, *J* = 1.2 Hz, H-1⁗), 5.41 (1H, d, *J* = 7.6 Hz, H-1″), 5.23 (1H, m, H-24), 5.12 (1H, d, *J* = 7.6 Hz, H-1‴), 4.97 (1H, d, *J* = 7.2 Hz, H-1′), 4.33 (1H, m, H-6), 4.12 (1H, m, H-12), 3.59 (1H, d, *J* = 8.0 Hz, H-3), 1.99 (3H, s, H-28), 1.64 (3H, s, H-27), 1.61 (3H, s, H-26), 1.61 (3H, s, H-21), 1.59 (3H, s, H-29), 1.09 (3H, s, H-18), 0.98 (3H, s, H-30), 0.93 (3H, s, H-19); ^13^C NMR (100 MHz, pyridine-*d*_5_) δ (ppm), see Table 3; ESI-MS *m*/*z* 1118 ([M + Na]^+^).

**Table 3:** ^13^C NMR Spectral Data of **18** (Recorded in Pyridine-*d*_*5*_ at 100 MHz)

Position	δ (ppm)	Position	δ (ppm)
1	39.2	3-*O*-sugar	glc
2	26.7	1	105.4
3	89.5	2	83.4
4	40.7	3	78.1
5	61.8	4	71.8
6	67.6	5	78.2
7	47.5	6	62.9
8	41.2	3-*O*-sugar	(2–1)glc
9	49.7	1	106.0
10	38.7	2	77.0
11	30.8	3	78.5
12	70.3	4	71.7
13	49.1	5	78.2
14	51.7	6	62.9
15	30.8	20-*O*-sugar	glc
16	25.9	1	98.1
17	51.4	2	75.1
18	17.9	3	79.3
19	17.4	4	72.1
20	83.4	5	76.6
21	22.4	6	68.5
22	36.2	20-*O*-sugar	(6–1)ara
23	23.2	1	110.2
24	126.1	2	83.4
25	131.1	3	78.9
26	25.9	4	86.0
27	17.5	5	62.7
28	31.4		
29	16.8		
30	17.6		

Panaxjapyne D (**21**)**:** colorless syrup; [α]_*D*_^25^ + 17 (*c* 0.05, MeOH); UV (MeOH) λ _max_ (log ε) 281 (1.3), 266 (1.4), 252 (1.2), 239 (1.0), 212 (1.9) nm; IR (neat) ν_max_ 3325, 2924, 2855, 2234, 1724, 1627, 1072, 1038 cm^–1^; ^1^H NMR (500 MHz, CD_3_OD) δ (ppm) 6.13 (1H, dq, *J* = 11.0, 7.0 Hz, H-9), 5.56 (1H, dq, *J* = 11.0, 1.5 Hz, H-8), 4.65 (1H, d, *J* = 8.5 Hz, H-1′), 4.41 (1H, d, *J* = 7.0 Hz, H-1″), 3.99 (1H, m, H-1a), 3.72 (1H, m, H-1b), 2.52 (1H, t, *J* = 7.0 Hz, H-3), 1.88 (1H, dd, *J* = 7.0, 1.5 Hz, H-10), 1.84 (2H, m, H-2); ^13^C NMR (125 MHz, CD_3_OD) δ (ppm) 141.5 (C-9), 108.6 (C-8), 103.8 (C- 1″), 101.6 (C-1′), 83.8 (C- 7), 81.6 (C-2′), 78.1 (C-6), 76.8 (C-3′′), 76.5 (C-5′′), 76.4 (C-5′), 76.3 (C-3′), 74.7 (C-2′′), 71.1 (C-5), 70.0 (C-4′), 69.9 (C-4′′), 67.9 (C-1), 64.3 (C-4), 61.3 (C-6′), 61.2 (C-6′′), 28.3 (C-2), 15.5 (C-3), 14.9 (C-10); ESI-MS *m*/*z* 495 ([M + Na]^+^); HR-ESI-MS *m*/*z* 495.1840 [M + Na]^+^ (calcd. for C_22_H_32_O_11_Na 495.1842).

Panaxjapyne E (**22**)**:** colorless syrup; [α]_*D*_^25^ + 270 (*c* 0.06, MeOH); UV (MeOH) (log ε) λ_max_: 258 (2.5), 245 (2.7), 232 (2.7), 203 (3.5) nm; IR (neat) ν_max_ 3340, 2931, 2858, 2261, 1786, 1639, 1080 cm^–1^; ^1^H NMR (400 MHz, CDCl_3_) δ (ppm) 5.82 (1H, ddq, *J* = 16.4, 10.0, 7.2 Hz, H-16), 5.02 (1H, dd, *J* = 16.4, 2.0 Hz, H-17a), 4.93 (1H, dd, *J* = 10.0, 2.0 Hz, H-17b), 4.52 (1H, d, *J* = 2.8 Hz, H-3), 4.16 (1H, d, *J* = 2.8 Hz, H-8), 3.90 (2H, m, H-1), 3.76 (1H, m, H-2), 3.52 (1H, m, H-11), 3.50 (3H, s, OCH_3_), 3.25 (1H, dd, *J* = 7.2, 4.0 Hz, H-9), 2.96 (1H, dd, *J* = 7.2, 4.0 Hz, H-10), 2.08 (2H, m, H-15), 1.76 (2H, m, H-12), 1.57 (2H, m, H-13), 1.45 (2H, m, H-14); ^13^C NMR (100 MHz, CDCl_3_) δ (ppm) 138.8 (C-16), 114.5 (C-17), 77.9 (C-4), 75.9 (C-7), 74.3 (C-2), 71.7 (C-6), 70.5 (C-8), 70.2 (C-5), 69.6 (C-11), 63.9 (C-3), 63.0 (C-1), 59.3 (C-10), 56.9 (C-9), 56.9 (OCH_3_), 34.6 (C-12), 33.7 (C-15), 28.9 (C-13), 24.6 (C-14); ESI-MS *m*/*z* 361 ([M + Na]^+^).

Panaxjapyne F (**23**)**:** colorless syrup; [α]_*D*_^25^ + 17 (*c* 0.05, MeOH); UV (MeOH) (log ε) λ_max_: 281 (1.3), 266 (1.4), 252 (1.2), 239 (1.0), 212 (1.9) nm; IR (neat) ν_max_ 3325, 2924, 2855, 2234, 1724, 1627, 1072, 1038 cm^–1^; ^1^H NMR (400 MHz, CDCl_3_) δ (ppm) 4.51 (1H, br d, *J* = 2.0, H-3), 4.23 (1H, d, *J* = 7.2 Hz, H-8), 3.86 (2H, m, H-1), 3.74 (1H, m, H-2), 3.57 (1H, m, H-11), 3.47 (3H, s, OCH_3_), 3.24 (1H, dd, *J* = 7.2, 4.0 Hz, H-9), 2.97 (1H, dd, *J* = 7.2, 4.0 Hz, H-10), 1.61 (2H, m, H-12), 1.52–1.41 (8H, m, H-13, H-14, H-1, H-16), 0.88 (1H, t, *J* = 6.4 Hz, H-17); ^13^C NMR (100 MHz, CDCl_3_) δ (ppm) 78.3 (C-4), 75.9 (C-7), 74.6 (C-2), 71.7 (C-6), 70.2 (C-8), 69.9 (C-5), 69.4 (C-11), 63.6 (C-3), 62.9 (C-1), 59.5 (C-10), 57.0 (C-9), 57.0 (OCH_3_), 34.7 (C-12), 31.8 (C-16), 29.3 (C-14), 25.1 (C-13), 22.6 (C-15), 14.1 (C-17); ESI-MS *m*/*z* 363 ([M + Na]^+^).

### Bioactivity Examination

The relaxation activity of corpus cavernosum of rats were examined according to the previously reported method.^48^ The male Sprague–Dawley rats (250–300 g) were used and housed in a light-controlled room with a 12 h day/night cycle and given free access to food and water. Experiments were approved by the Animal Care Committee of the National Research Institute of Chinese Medicine (No. 97-P-06, 10/22/2008). Tissue preparation and endothelium disruption were performed as described.^48^ Briefly, after equilibration, the cavernosal strip contractions were evoked by PE (3 μM) or KCl (40 mM). When the contractile response was stabilized, the tested compounds were added to examine the relaxation against different PE doses. The relaxation induced was expressed as a percentage of relaxation against PE-, KCl-evoked contractions running from 0 to 100% and the EC_50_ was calculated by PCS 4.0 software (Pharmacological Calculation System, Springer-Verlag, New York, NY, USA).

### Molecular Docking Study

The *in silico* evaluation was conducted on AutoDock Vina software.^49^ The crystal structure of the 3′,5′ -cyclic phosphodiesterase enzyme (PDE5) and its cocrystallized ligand vardenafil has been characterized,^50^ and a. PDB file was downloaded from the Protein Databank (PDB ID: 1XP0). Vardenafil (a clinically approved PDE5 inhibitor) was first computed to determine the accuracy of the present docking model. The 3D structures of the ligands were constructed in the Chem3D program. The hydrogen supplement, Gasteiger charge measurement for protein atoms, and selection of flexible torsions for ligands were conducted by AutodockTools (ADT ver. 1.5.6). The size of the grid was designed by ligand size and a grid center at dimensions (*x*, *y*, and *z*, respectively): – 22.4, 28.6, 62.7 was determined. The binding affinity energy was provided as docking scores and is shown in kcal/mol. The best interaction was considered to be only the top-scoring pose. The visualization of the best docking interactions was analyzed in Biovia Discovery Studio client 2021.^51^

## Supporting Information Available

The MS/HRMS, UV, IR, ^1^H, ^13^C, and 2D NMR spectra, including the detailed HMBC correlations for all the undescribed compounds **18**, **21**, **22**, and **23** (PDF)
