# Supplementary material for: Aphrodisiac Principles and other Constituents from the Roots of Panax quinguefolium and Panax ginseng
Source: ACS Omega. 2024 Jul 18;9(30):33239–50. doi: 10.1021/acsomega.4c04965 (PMC11292828; doi:10.1021/acsomega.4c04965)

Supporting Information

Aphrodisiac Principles and other Constituents from the Roots of *Panax quinguefolium* and *Panax ginseng*

Kun-Ching Cheng [a,#], Hsiu-Hui Chan [b,#], Wen-Fei Chiou [c], Chia-Hung Wu [d], Yue-Chiun Li [e], Hao-Ze Li [e], Ping-Chung Kuo [e,*], and Tian-Shung Wu [e,*]

[a] Taiwan Sugar Research Institute, Tainan 70176, Taiwan

[b] Department of Chemistry, National Cheng Kung University, Tainan 701, Taiwan

[c] National Research Institute of Chinese Medicine, Taipei 112, Taiwan

[d] School of Post-Baccalaureate Chinese Medicine, China Medical University, Taichung 404, Taiwan

[e] School of Pharmacy, College of Medicine, National Cheng Kung University, Tainan 701, Taiwan

[#] Both authors contributed equally to this work.

Corresponding Authors

Prof. Tian-Shung Wu.

E-mail address: tswu@mail.ncku.edu.tw

Prof. Ping-Chung Kuo

E-mail address: z10502016@ncku.edu.tw

Contents

Figure S1. Fractionation of the *P. quinquefolium* and *P. ginseng* root extracts and the EC_{50} values of relaxation of corpus cavernosum of rats.

Figure S2. Purification of the compounds **1-6** from the bioactive fractions of *P. quinquefolium* and *P. ginseng* root extracts.

Figure S3. Purification of the compounds **1, 2, 5, 7-34** from the ethyl acetate layer of *P. quinquefolium* root extracts.

Figure S4. ESI-MS of compound **18**.

Figure S5. IR spectrum of compound **18**.

Figure S6. ^{1}H-NMR of compound **18**.

Figure S7. ^{13}C-NMR of compound **18**.

Figure S8. COSY of compound **18**.

Figure S9. NOESY of compound **18**.

Figure S10. HMQC of compound **18**.

Figure S11. HMBC of compound **18**.

Figure S12. UV spectrum of compound **21**.

Figure S13. UV spectrum of compound **22**.

Figure S14. UV spectrum of compound **23**.

Figure S15. IR spectrum of compound **21**.

Figure S16. IR spectrum of compound **22**.

Figure S17. IR spectrum of compound **23**.

Figure S18. HR-ESI-MS of compound **21**.

Figure S19. ^{1}H-NMR of compound **21**.

Figure S20. ^{13}C-NMR of compound **21**.

Figure S21. HMQC of compound **21**.

Figure S22. COSY of compound **21**.

Figure S23. HMBC of compound **21**.

Figure S24. NOESY of compound **21**.

Figure S25. ESI-MS of compound **22**.

Figure S26. ^{1}H-NMR of compound **22**.

Figure S27. ^{13}C-NMR of compound **22**.

Figure S28. HMQC of compound **22**.

Figure S29. COSY of compound **22**.

Figure S30. HMBC of compound **22**.

Figure S31. NOESY of compound **22**.

Figure S32. ^{1}H-NMR of compound **23**.

Figure S33. ^{13}C-NMR of compound **23**.

Figure S34. ESI-MS of compound **23**.

Figure S35. HMQC of compound **23**.

Figure S36. COSY of compound **23**.

Figure S37. HMBC of compound **23**.

Figure S38. NOESY of compound **23**.

Figure S39. Basic structures of naturally purified triterpenoid saponins.

Figure S40. Biosynthetic pathway of triterpenoids.

Figure S41. Biosynthetic pathway of dammarenediol-type triterpenoids.

Figure S42. Biosynthetic pathway of protopanaxatriol, panaxatriol, and cotillol types triterpenoids.

Figure S43. Biosynthetic pathway of oleanolic acid type ginsenosides.

Figure S1. Fractionation of the *P. quinquefolium* and *P. ginseng* root extracts and the EC_{50} values of relaxation of corpus cavernosum of rats.

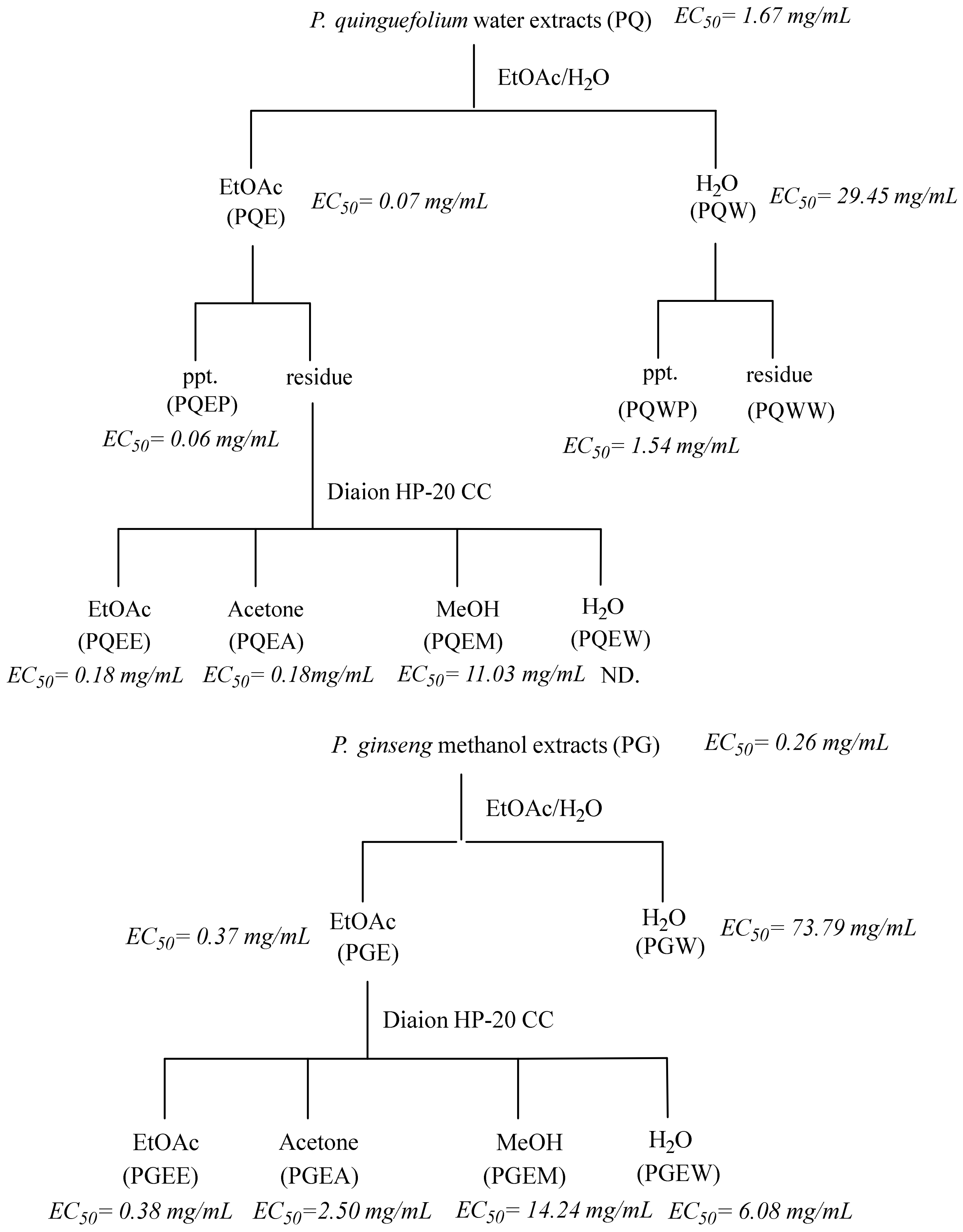

Figure S2. Purification of the compounds **1-6** from the bioactive fractions of *P. quinquefolium* and *P. ginseng* root extracts.

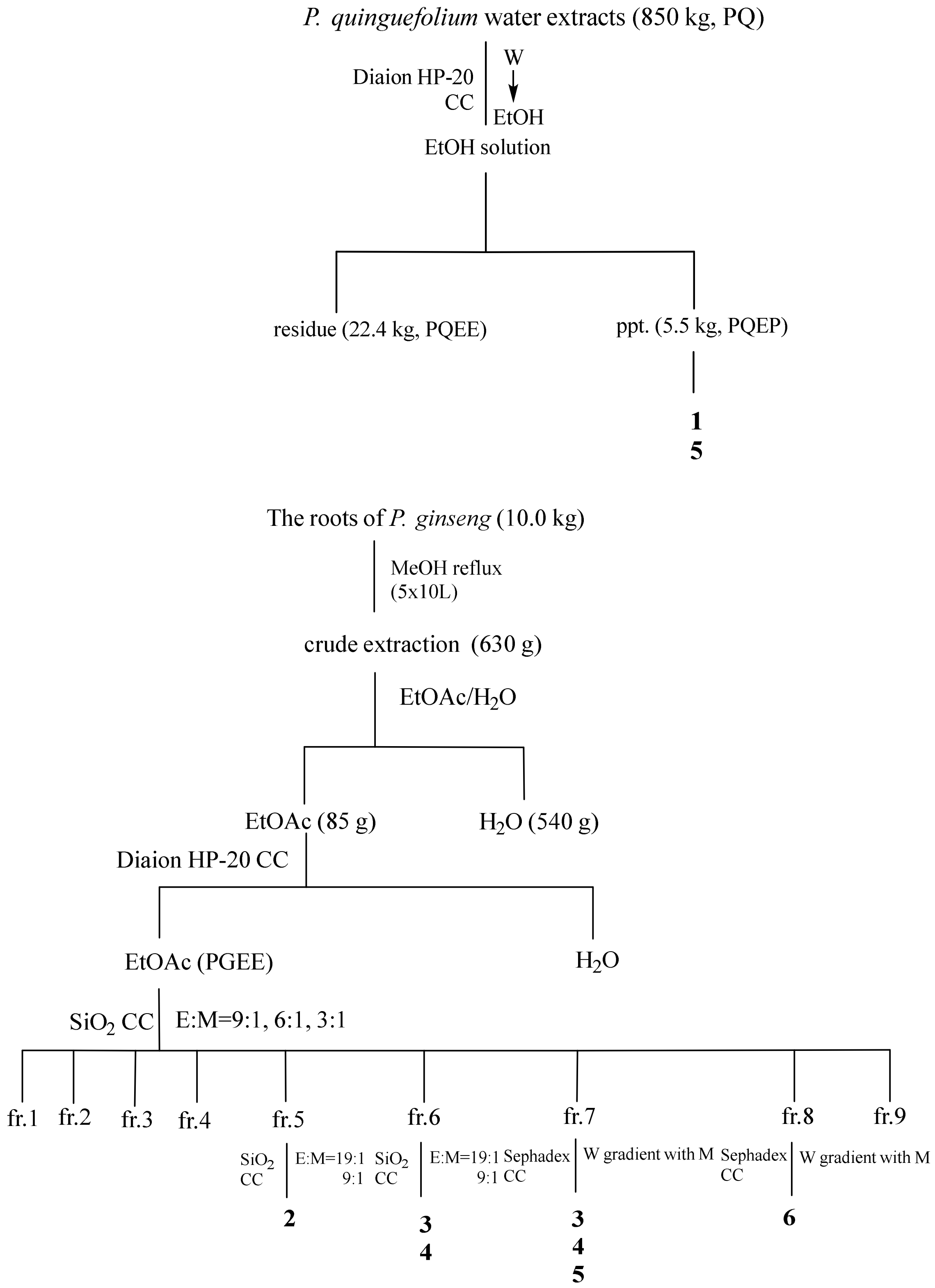

Figure S3. Purification of the compounds **1, 2, 5, 7-34** from the ethyl acetate layer of *P. quinquefolium* root extracts.

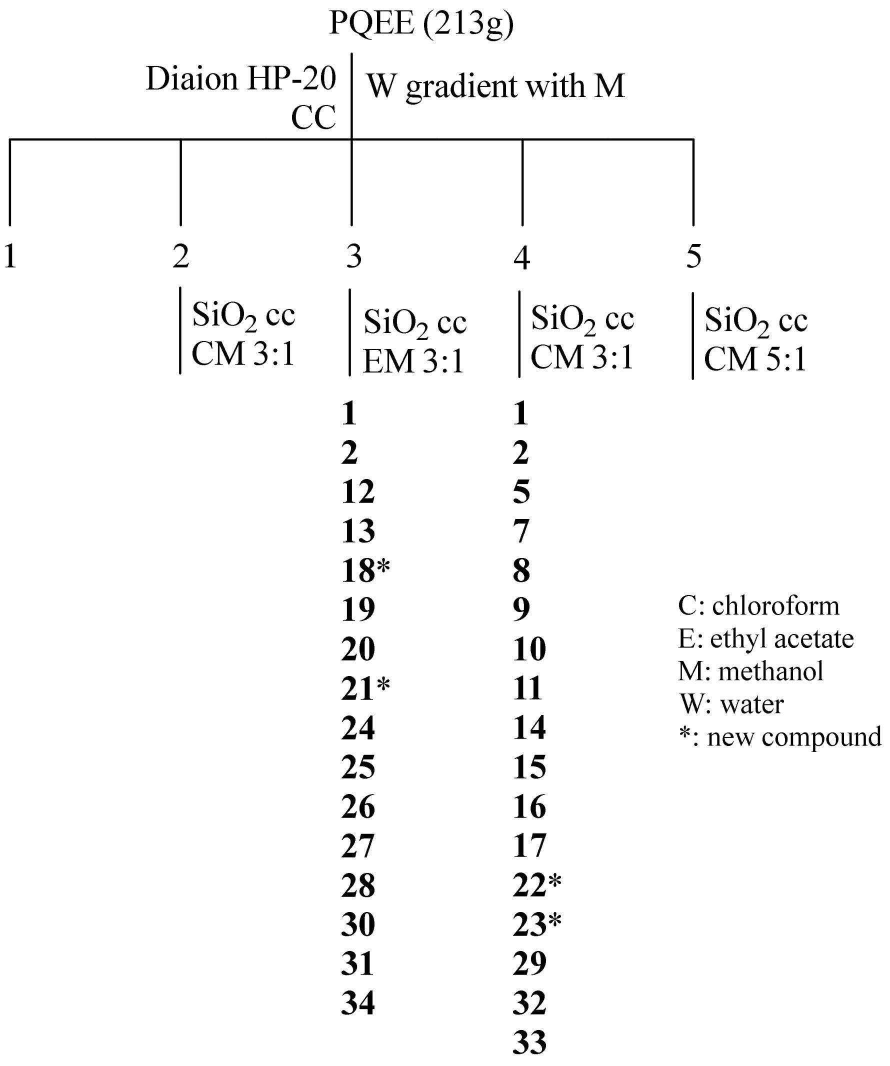

Figure S4. ESI-MS of compound **18**.

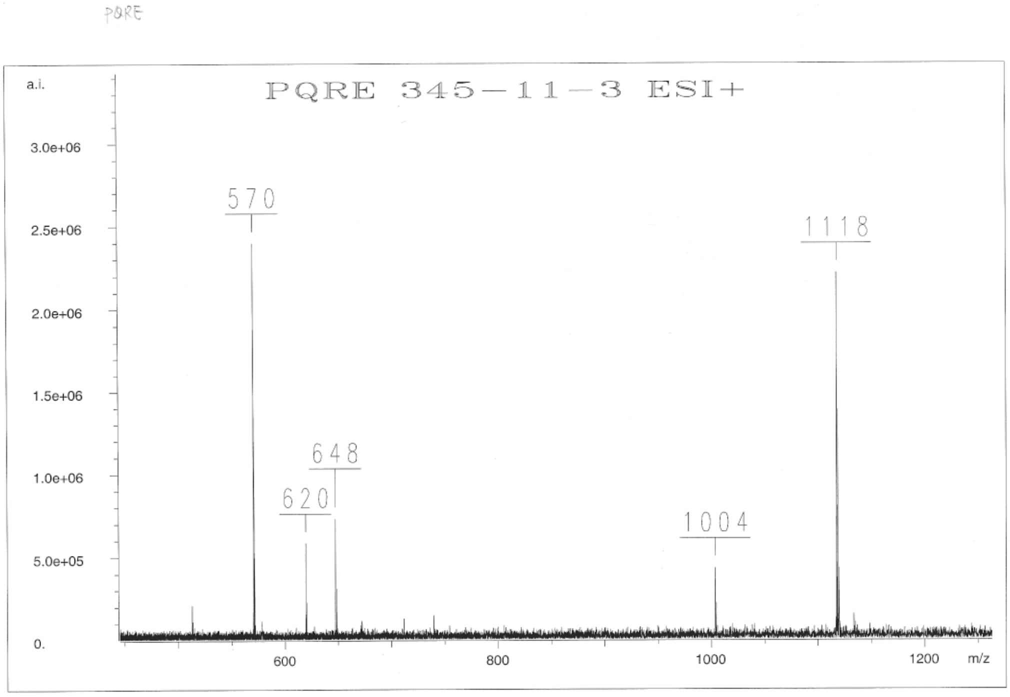

/d=/Data/yu/PQRE345113/2/pdata/1 Administrator Tue Feb 2 16:07:04 2010

Figure S5. IR spectrum of compound **18**.

SHIMADZU

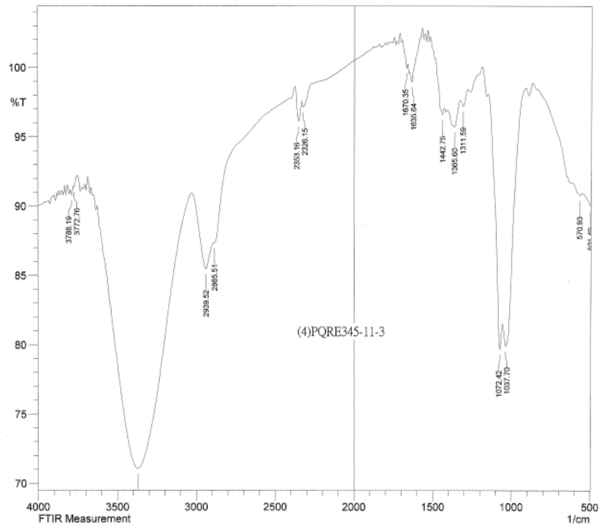

	Peak	Height	Corr. He	Base (H)	Base (L)	Area	Corr. Area
1	501.49	9.995	0.104	547.78	497.63	2.186	0.005
2	570.93	9.196	0.365	617.22	551.64	2.654	0.062
3	1037.7	20.084	3.381	1053.13	914.26	6.684	0.882
4	1072.4	20.319	4.601	1149.57	1056.99	5.093	0.612
5	1311.5	2.796	0.685	1327.03	1284.59	0.438	0.072
6	1365.6	4.278	0.413	1373.32	1330.88	0.641	0.045
7	2326.1	2.876	0.339	2330.01	2268.29	0.539	0.05
8	2353.1	3.889	1.972	2372.44	2333.87	0.534	0.2
9	2885.5	12.663	0.1	2889.37	2407.16	10.912	0.009
10	2939.5	14.532	3.101	3028.24	2893.22	7.778	0.99
11	3371.5	28.925	19.347	3626.17	3032.1	58.645	32.849
12	3772.7	8.63	0.177	3776.62	3749.62	0.996	0.009
13	3788.1	9.217	0.521	3795.91	3776.62	0.783	0.024
14	1442.7	3.421	0.429	1450.47	1431.18	0.27	0.017
15	1635.6	1.059	1.295	1651.07	1585.49	-0.097	0.156
16	1670.3	0.043	0.659	1697.36	1662.64	-0.118	0.045

CPMA12521;
FTIR Measurement

Date/Time; 2010/05/30 、U、 ㄜ01:57:29
No. of Scans;
Resolution;
Apodization;
User; Tswu1

Figure S6. ^{1}H-NMR of compound **18**.

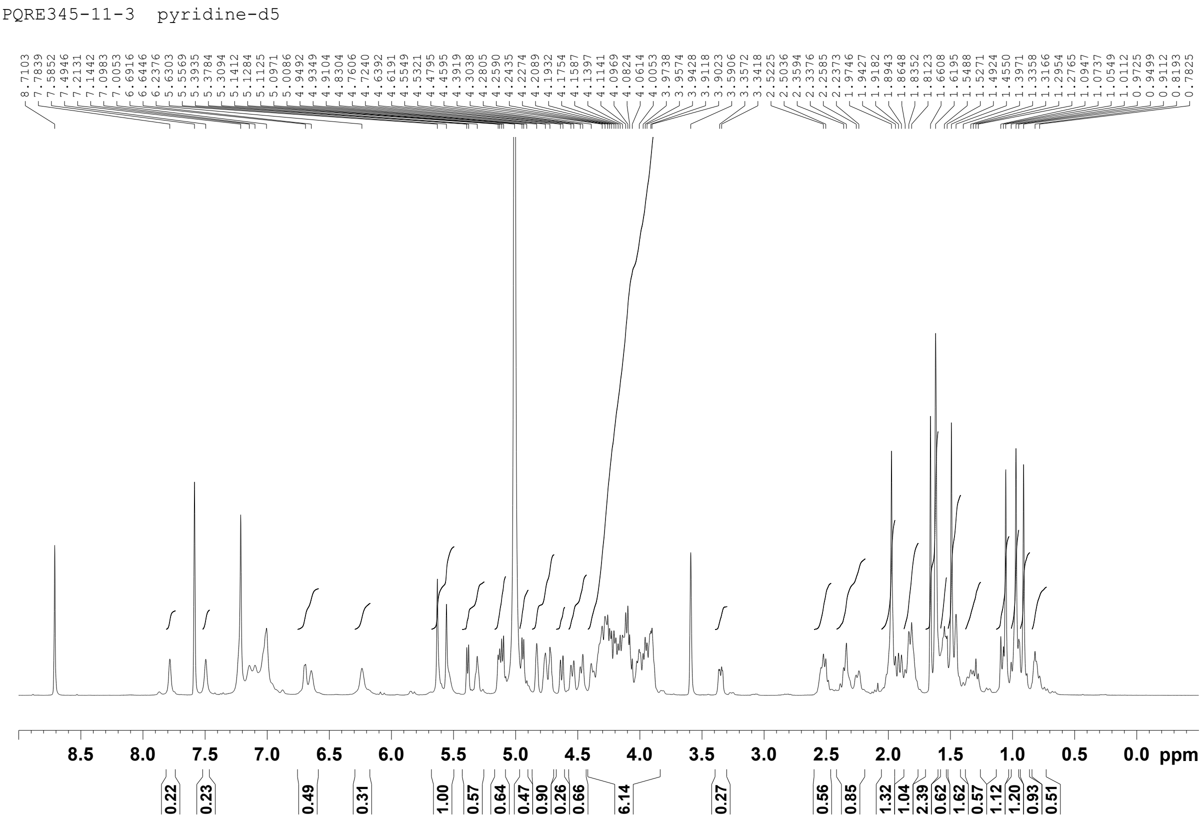

Figure S7. ^{13}C-NMR of compound **18**.

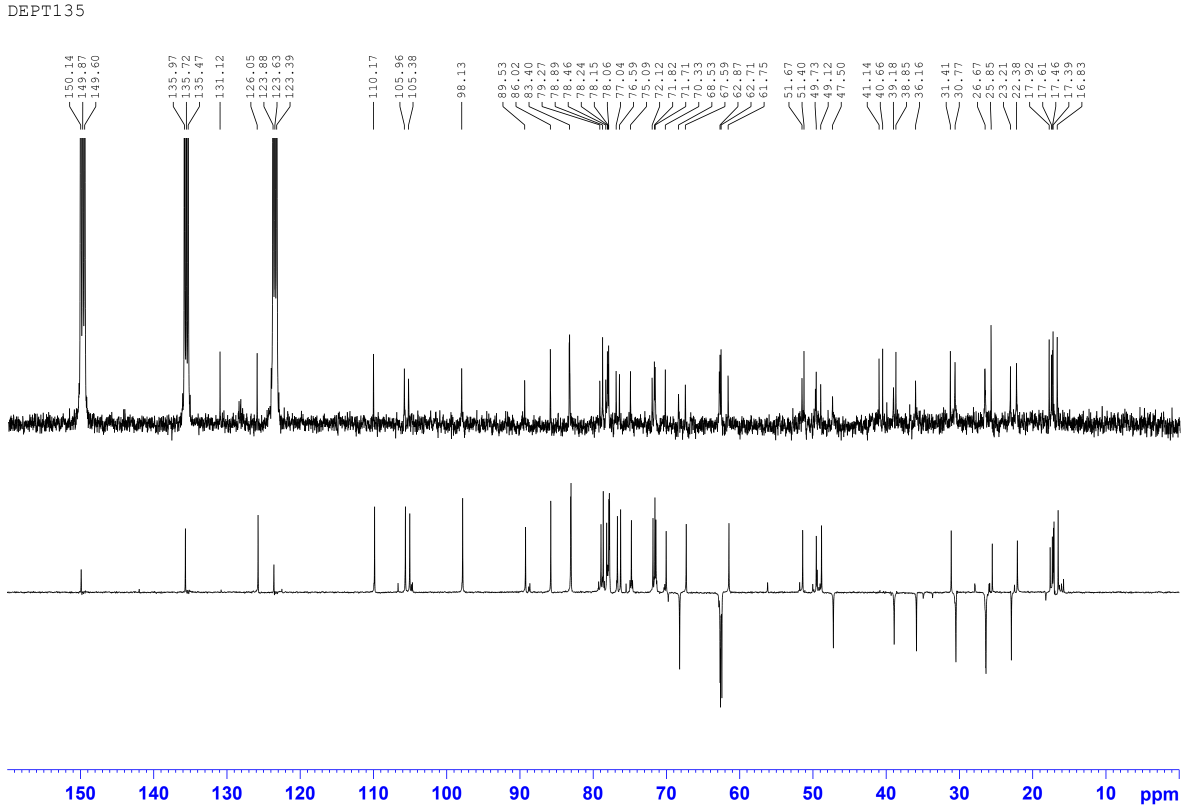

Figure S8. COSY of compound **18**.

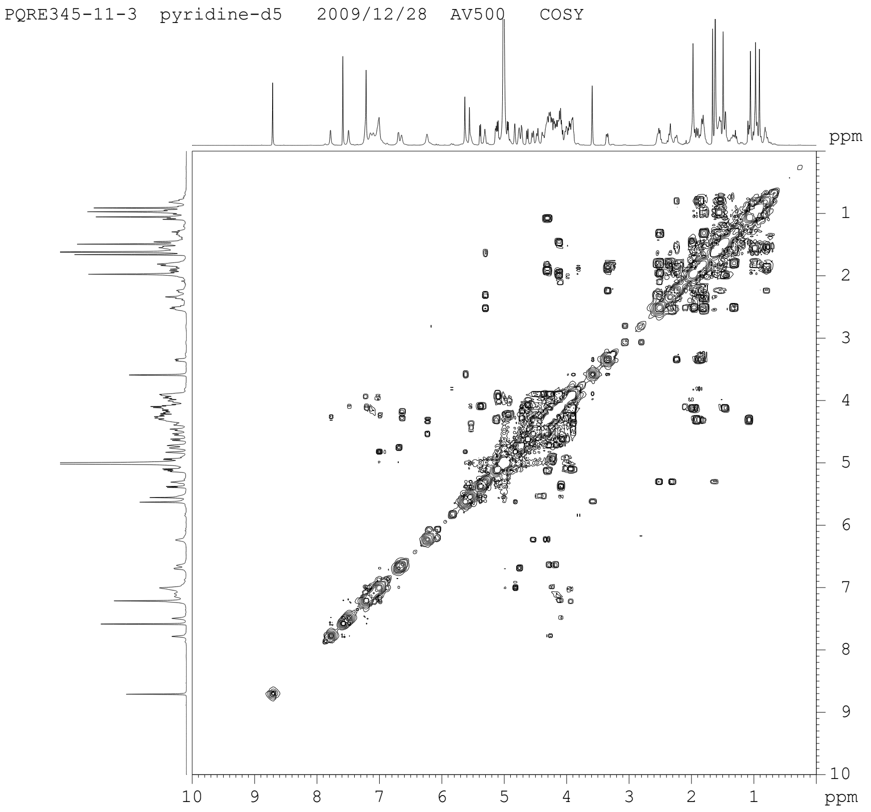

Figure S9. NOESY of compound **18**.

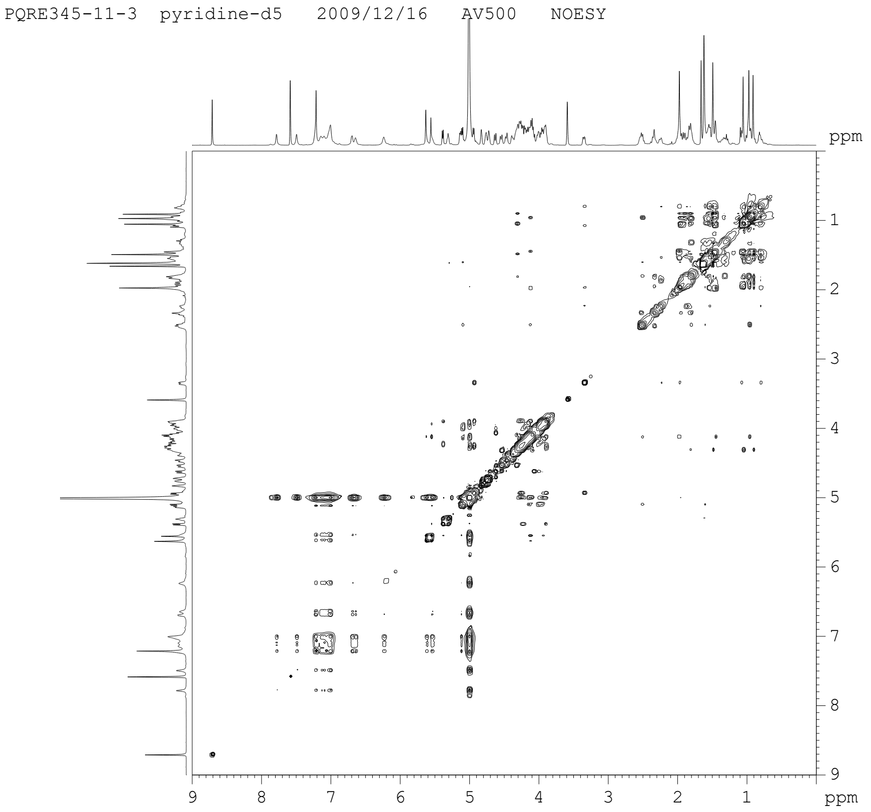

Figure S10. HMQC of compound **18**.

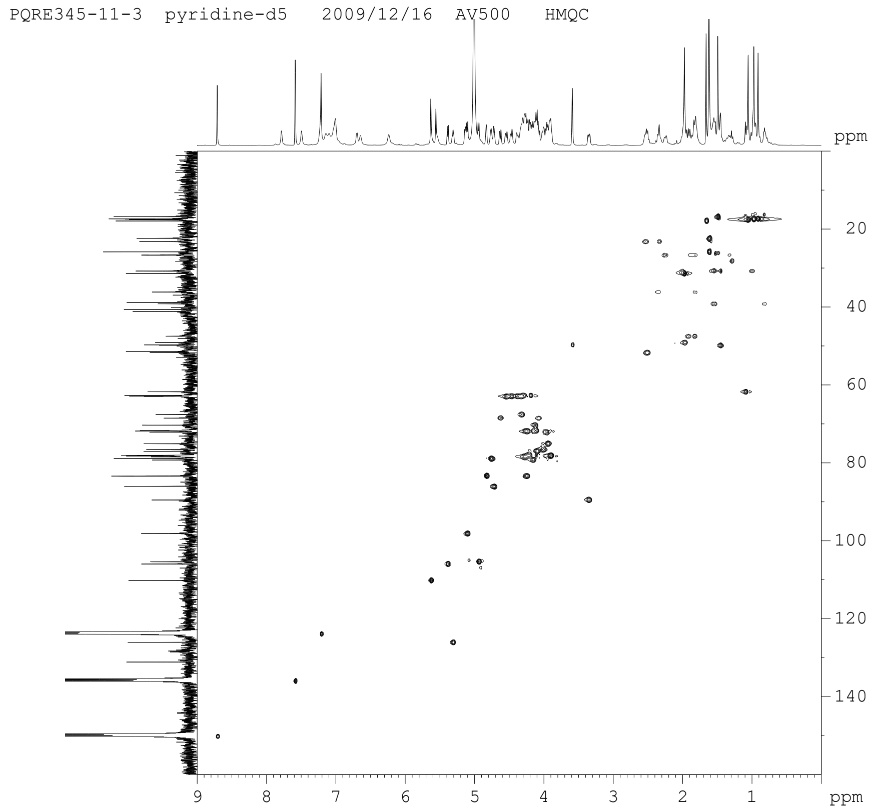

Figure S11. HMBC of compound **18**.

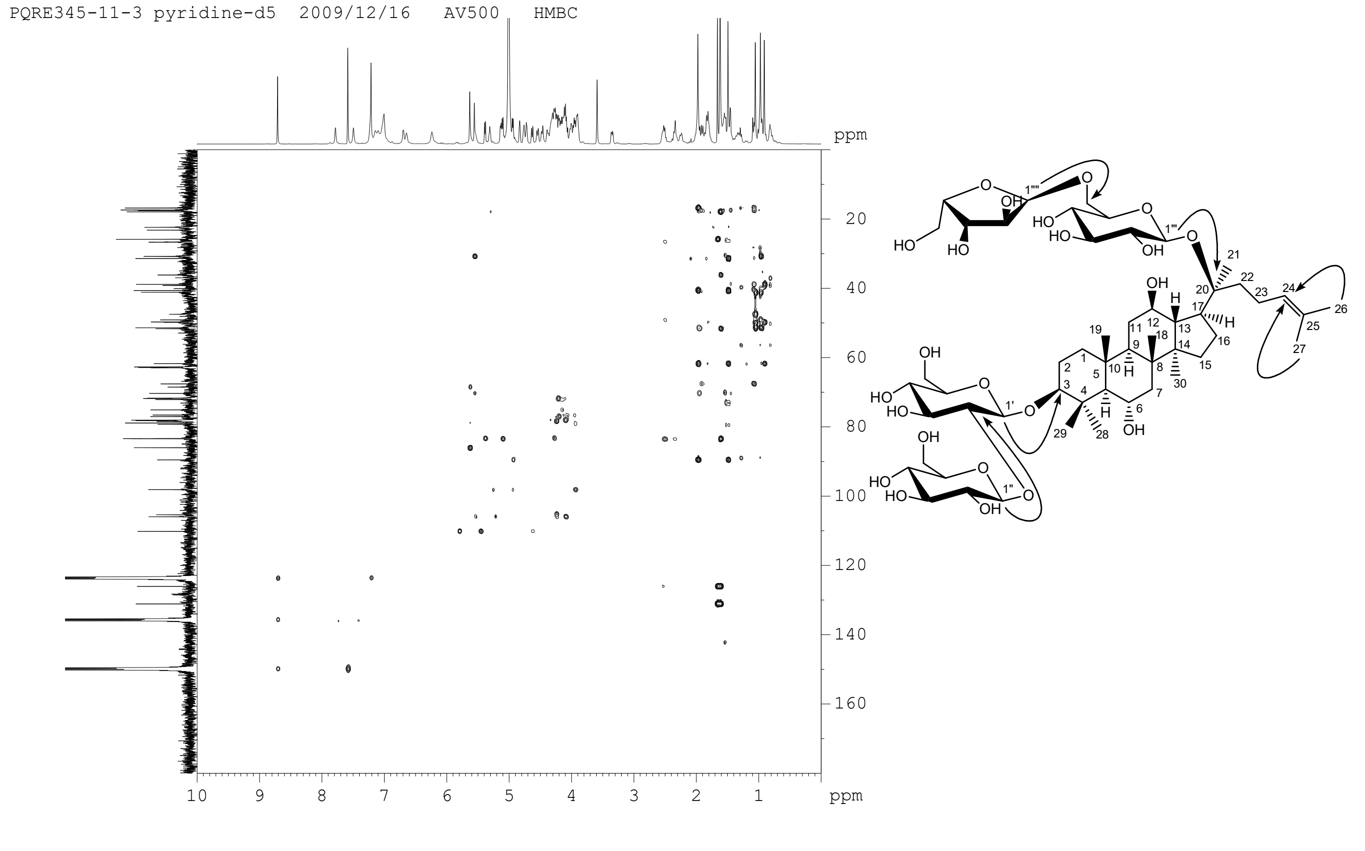

Figure S12. UV spectrum of compound **21**.

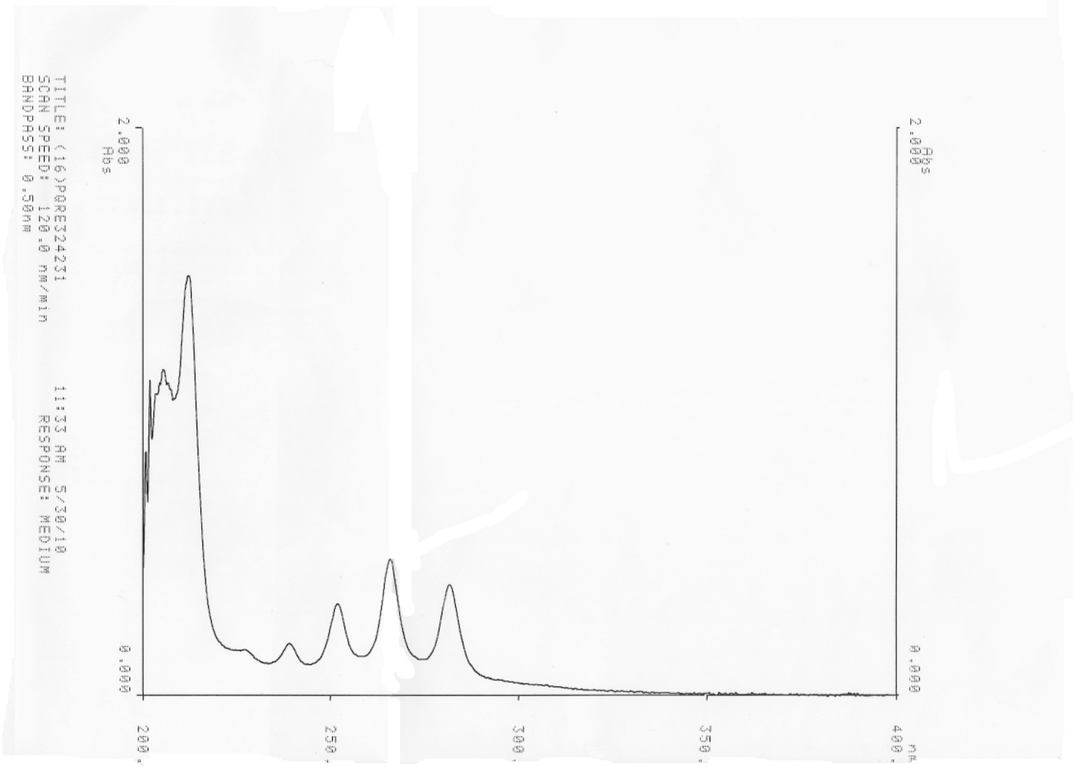

Figure S13. UV spectrum of compound **22**.

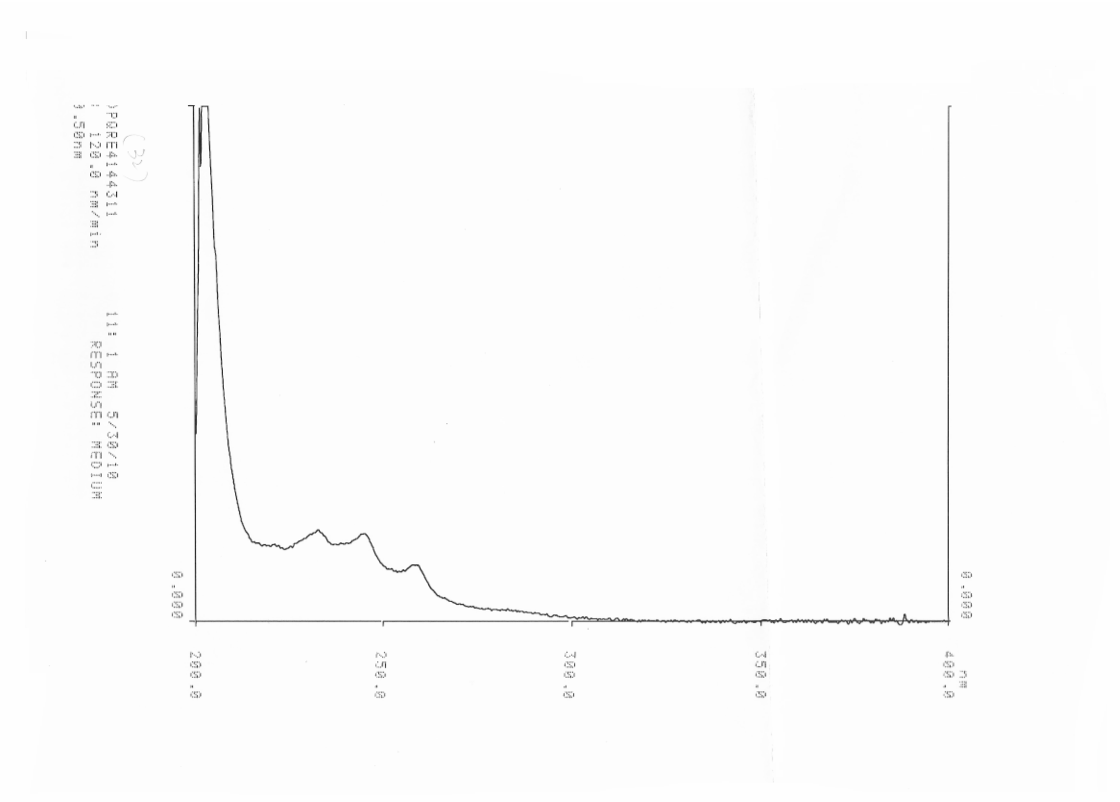

Figure S14. UV spectrum of compound **23**.

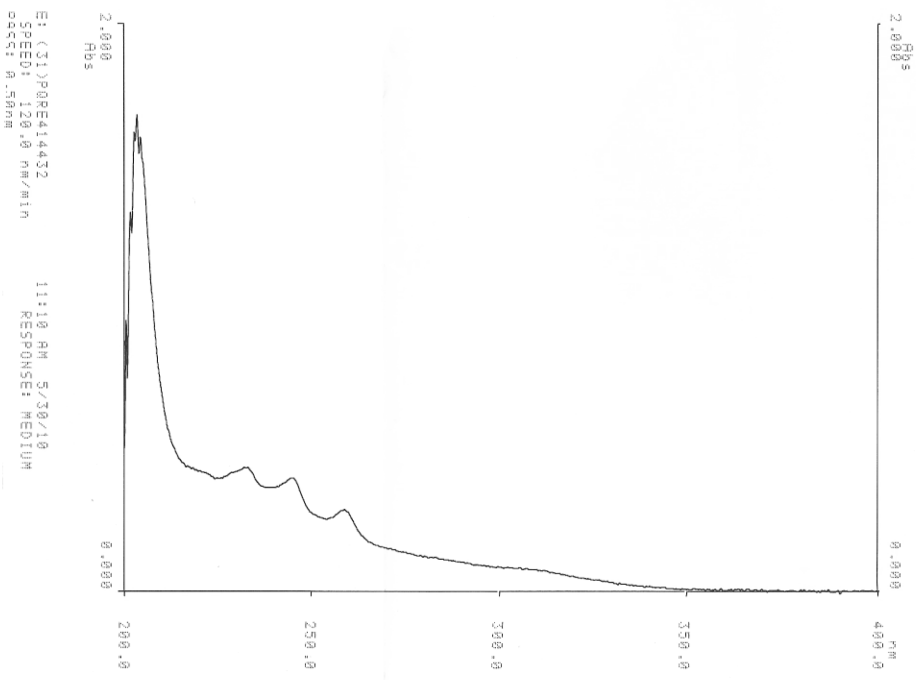

Figure S15. IR spectrum of compound **21**.

SHIMADZU

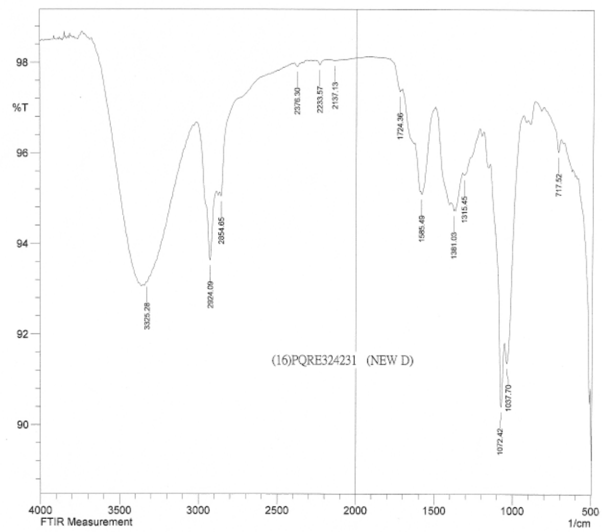

	Peak	Height	Corr. He	Base (H)	Base (L)	Area	Corr. Area
1	717.52	3.9719	0.3486	810.1	705.95	1.5615	0.0258
2	1037.7	8.6371	1.1448	1053.13	941.26	2.837	0.1847
3	1072.4	9.6001	2.0218	1149.57	1056.99	2.8531	0.2232
4	1315.4	4.4803	0.0909	1323.17	1219.01	1.8653	0.0442
5	1381.0	5.2759	0.3601	1400.32	1327.03	1.6201	0.0733
6	1585.4	4.9063	1.3899	1627.92	1504.48	2.2207	0.3748
7	1724.3	2.6452	0.1993	1786.08	1708.93	0.7771	0.0195
8	2854.6	4.9552	0.1604	2862.36	2588.47	3.505	0.0057
9	2924.0	6.373	1.9888	3012.81	2885.51	2.7368	0.4238
10	3325.2	6.8466	0.0458	3329.14	3035.96	6.7855	0.1447
11	2137.1	1.9683	0.0172	2156.42	2125.56	0.2651	0.0012
12	2233.5	2.0564	0.1046	2264.43	2206.57	0.5047	0.0092
13	2376.3	2.0924	0.0791	2391.73	2345.44	0.4159	0.0074

CPMA12521;
FTIR Measurement

Date/Time; 2010/05/30 、U、ｪ 02:22:34
No. of Scans; 3000
Resolution; 8 [1/cm]
Apodization; Happ-Genzel
User; Tswu1

Figure S16. IR spectrum of compound **22**.

SHIMADZU

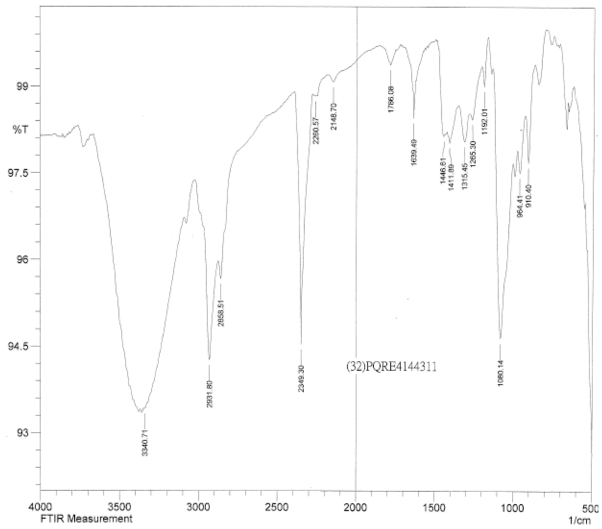

	Peak	Height	Corr. He	Base (H)	Base (L)	Area	Corr. Area
1	1080.1	5.3601	3.9292	1134.14	1010.7	2.0255	1.18
2	2858.5	4.3306	0.4282	2873.94	2553.75	3.1749	0.0294
3	3340.7	6.5793	0.0546	3344.57	3093.82	5.7101	0.2218
4	910.4	2.3202	1.1171	929.69	871.82	0.3596	0.0928
5	964.41	2.4971	0.5829	979.84	929.69	0.4522	0.0568
6	1192.0	1	0.5543	1207.44	1168.86	0.1021	0.0357
7	1265.3	1.5679	0.2703	1280.73	1207.44	0.3632	0.0238
8	1315.4	1.9574	0.5564	1354.03	1280.73	0.5305	0.0826
9	1411.8	1.9774	0.2931	1427.32	1354.03	0.5328	0.0349
10	1446.6	1.8582	0.1874	1481.33	1442.75	0.2377	0.0551
11	1639.4	1.5456	0.7505	1697.36	1627.92	0.2205	0.0384
12	1786.0	0.6281	0.2667	1816.94	1755.22	0.1361	0.039
13	2148.7	0.9257	0.1562	2183.42	2094.69	0.3177	0.0231
14	2260.5	1.1679	0.0158	2272.15	2252.86	0.0977	0.0007
15	2349.3	5.4653	4.3561	2391.73	2279.86	1.3366	0.7927
16	2931.8	5.7363	2.2497	3024.38	2873.94	2.7004	0.4891

CPMA12521;
FTIR Measurement

Date/Time; 2010/05/30 、U、ネ02:43:53
No. of Scans;
Resolution;
Apodization;
User; Tswu1

Figure S17. IR spectrum of compound **23**.

SHIMADZU

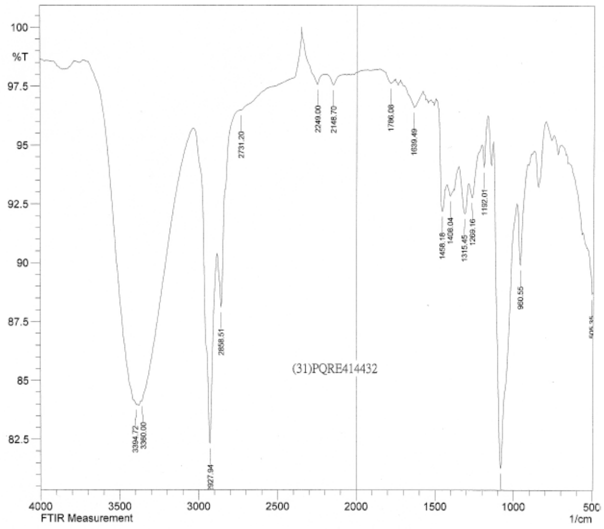

	Peak	Height	Corr. He	Base (H)	Base (L)	Area	Corr. Area
1	505.35	11.315	0.23	563.21	501.49	2.76	0.004
2	960.55	10.093	3.078	979.84	918.12	2.206	0.365
3	1080.1	18.717	12.933	1134.14	983.7	8.844	4.663
4	1269.1	7.225	0.98	1288.45	1207.44	2.216	0.13
5	1315.4	7.915	1.568	1354.03	1292.31	1.979	0.241
6	2731.2	3.526	0.019	2735.06	2534.46	2.636	0.004
7	2858.5	11.895	3.272	2881.65	2738.92	3.989	0.336
8	2927.9	17.679	9.56	3032.1	2885.51	6.904	2.246
9	3360	15.887	0.279	3367.71	3035.96	14.975	0.206
10	1192.0	5.944	1.464	1207.44	1168.86	0.839	0.092
11	1408.0	7.168	0.418	1427.32	1384.89	1.331	0.042
12	1458.1	7.816	2.113	1492.9	1442.75	1.337	0.22
13	1639.4	3.405	0.615	1674.21	1581.63	1.261	0.13
14	1786.0	2.378	0.303	1816.94	1762.94	0.532	0.045
15	2148.7	2.447	0.441	2198.85	2090.84	1.021	0.07
16	2249	2.43	1.099	2349.3	2198.85	1.151	0.492
17	3394.7	16.035	0.072	3410.15	3387	1.749	0.005

CPMA12521;
FTIR Measurement

Date/Time; 2010/05/30 、U、ネ02:41:56
No. of Scans;
Resolution;
Apodization;
User; Tswu1

Figure S18. HR-ESI-MS of compound **21**.

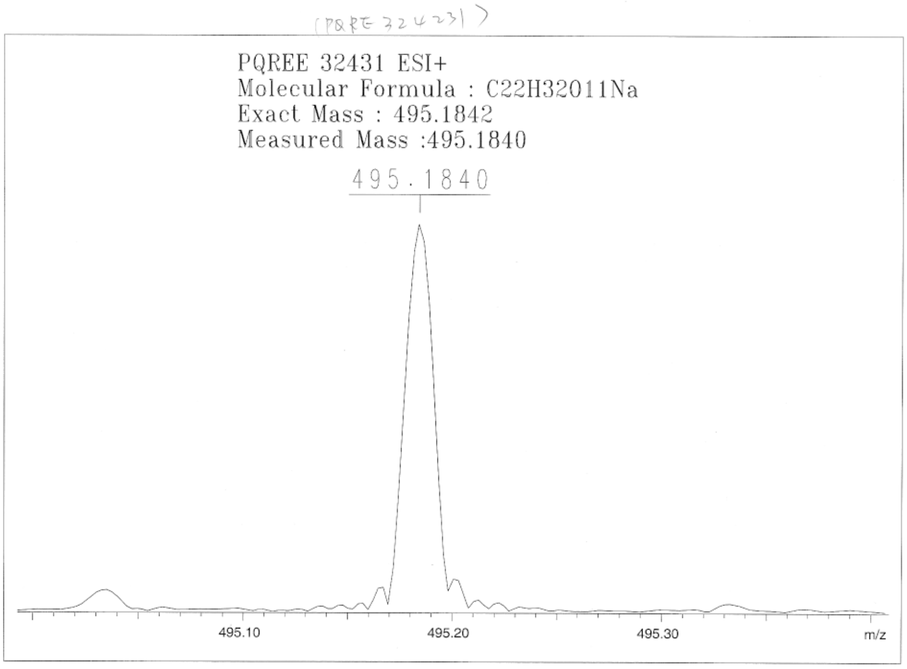

Figure S19. ^{1}H-NMR of compound **21**.

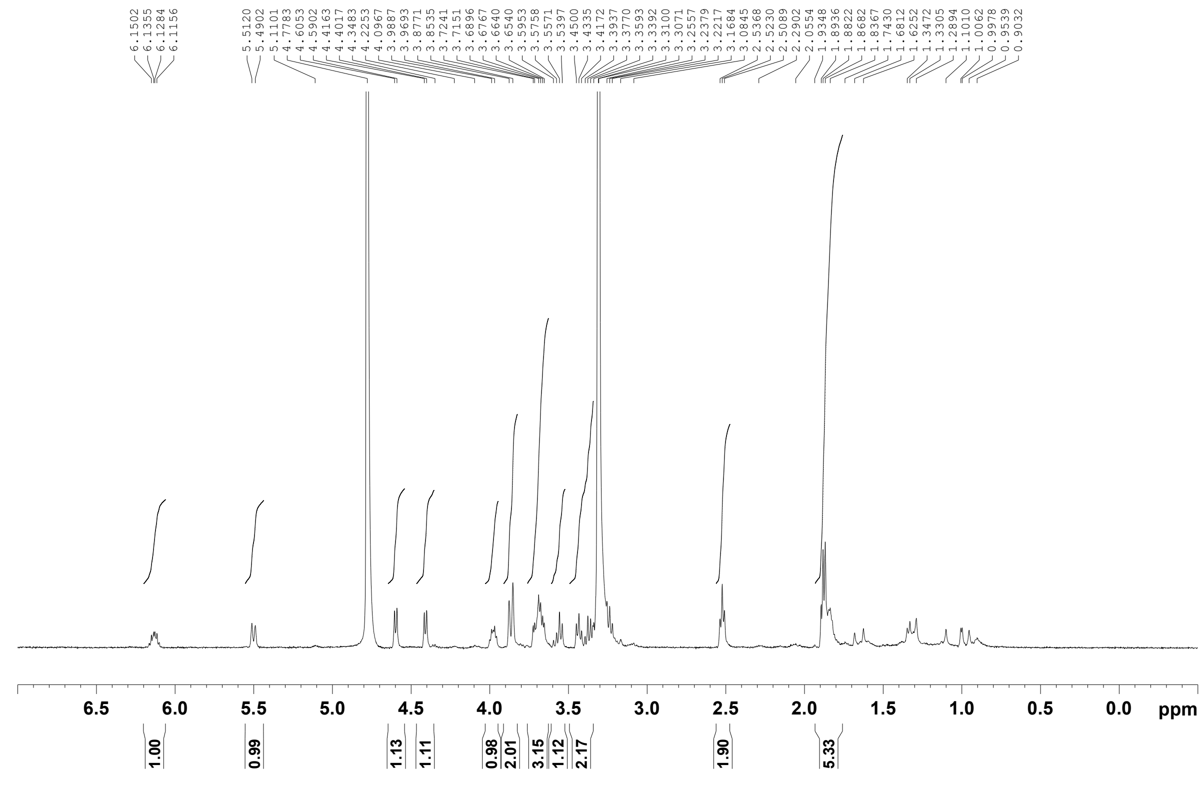

Figure S20. ^{13}C-NMR of compound **21**.

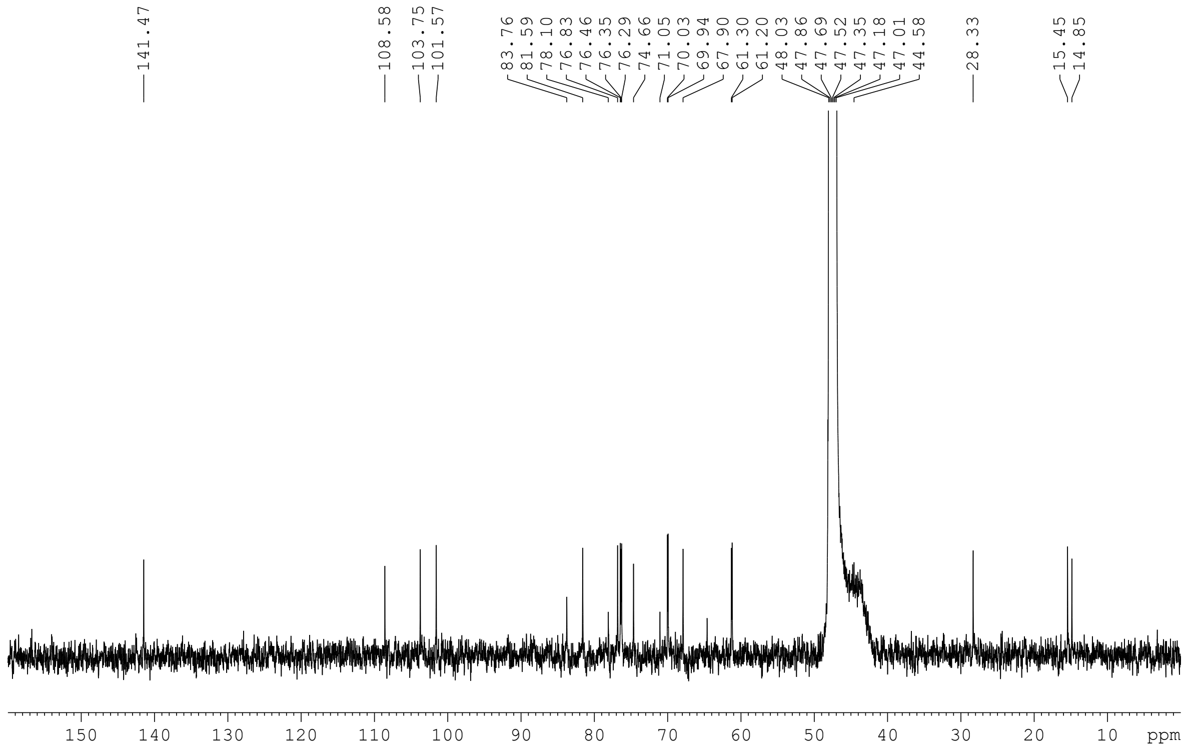

Figure S21. HMQC of compound **21**.

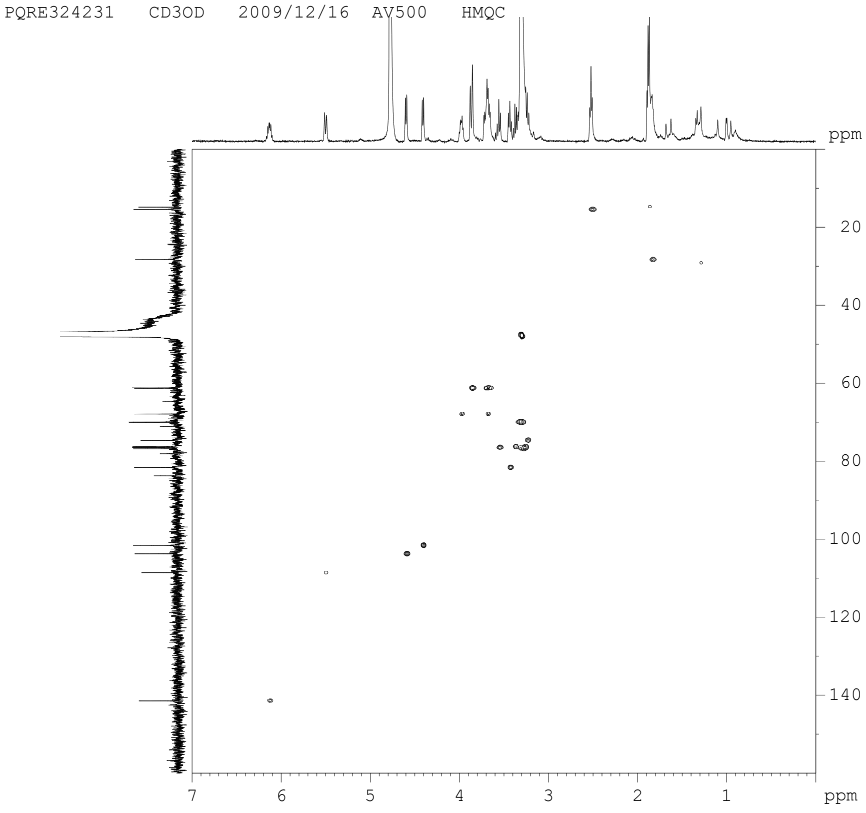

Figure S22. COSY of compound **21**.

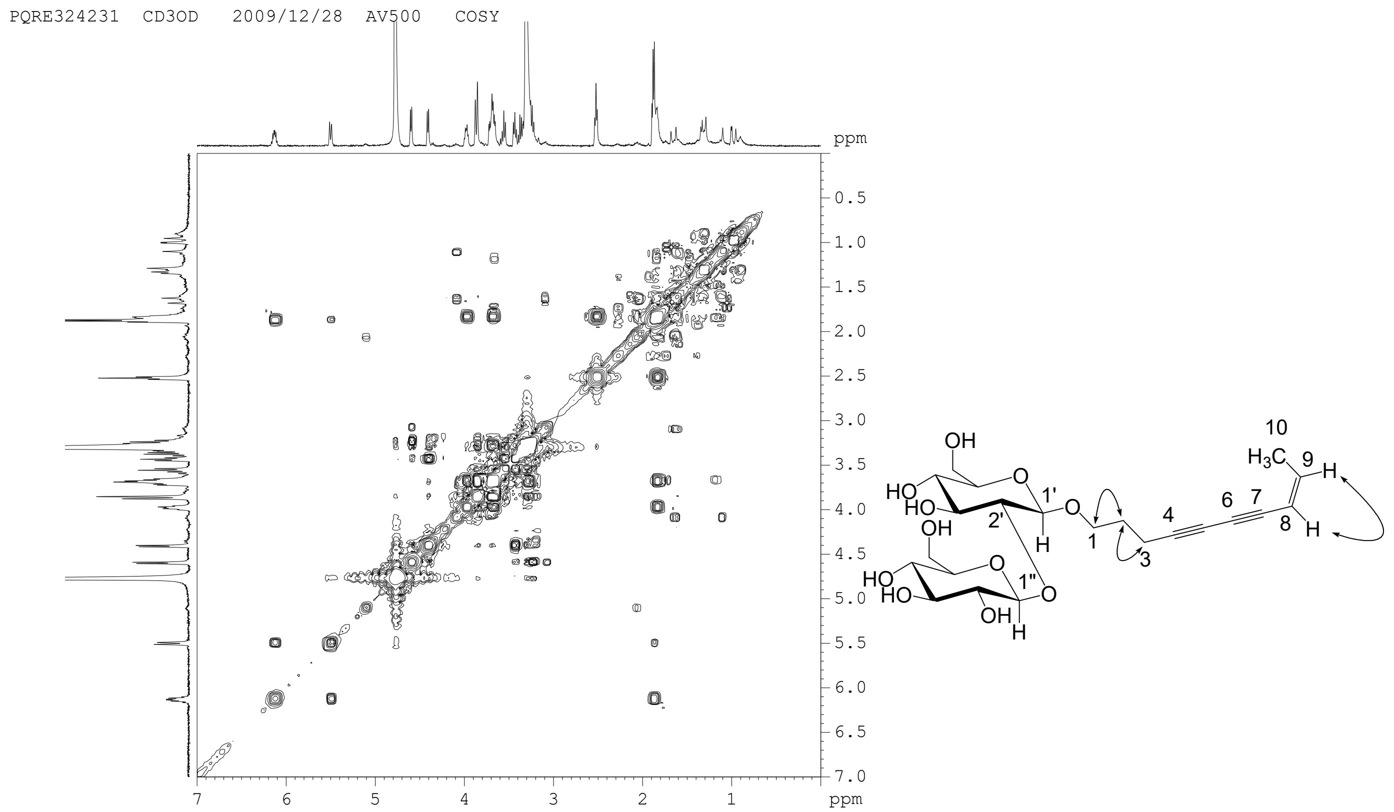

Figure S23. HMBC of compound **21**.

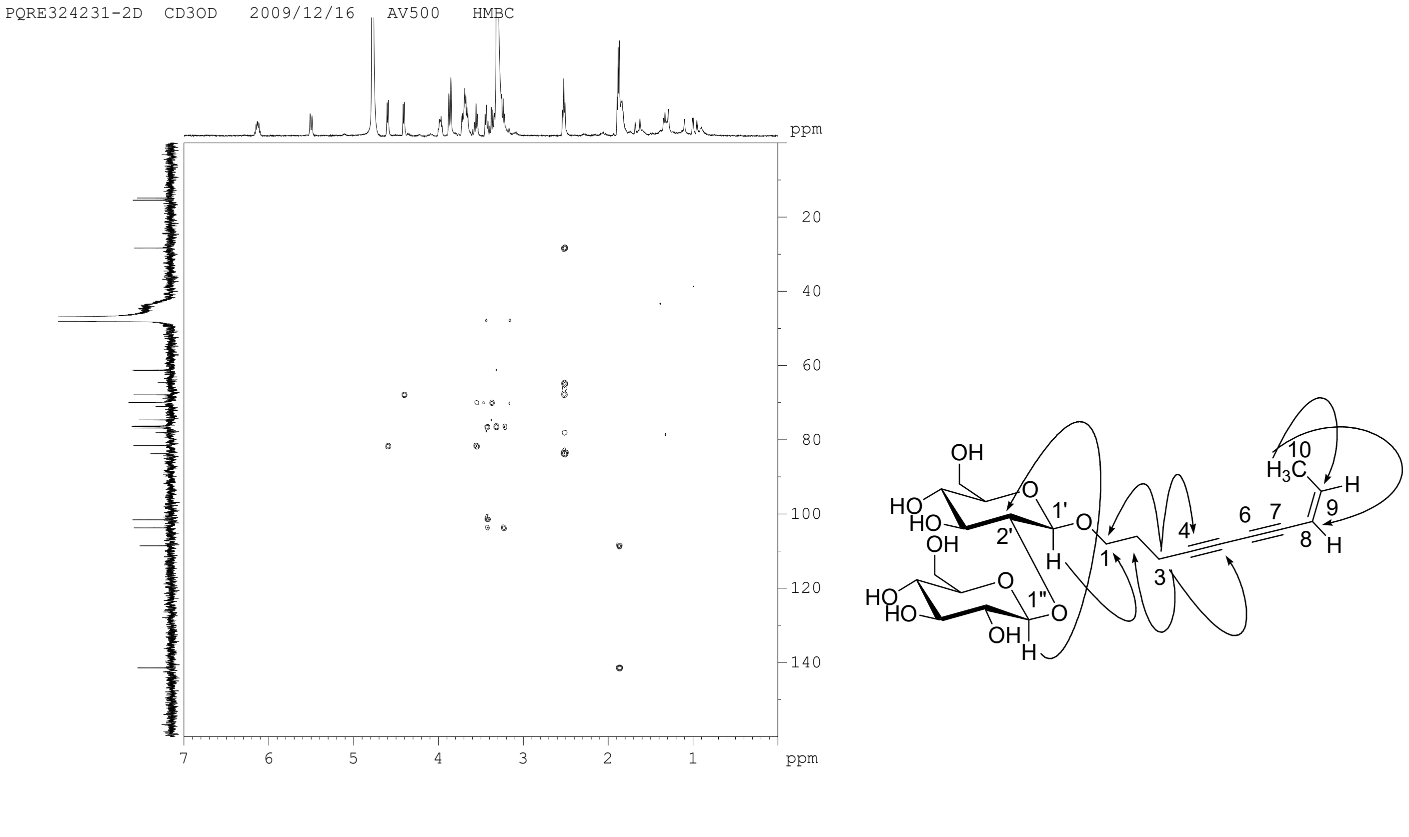

Figure S24. NOESY of compound **21**.

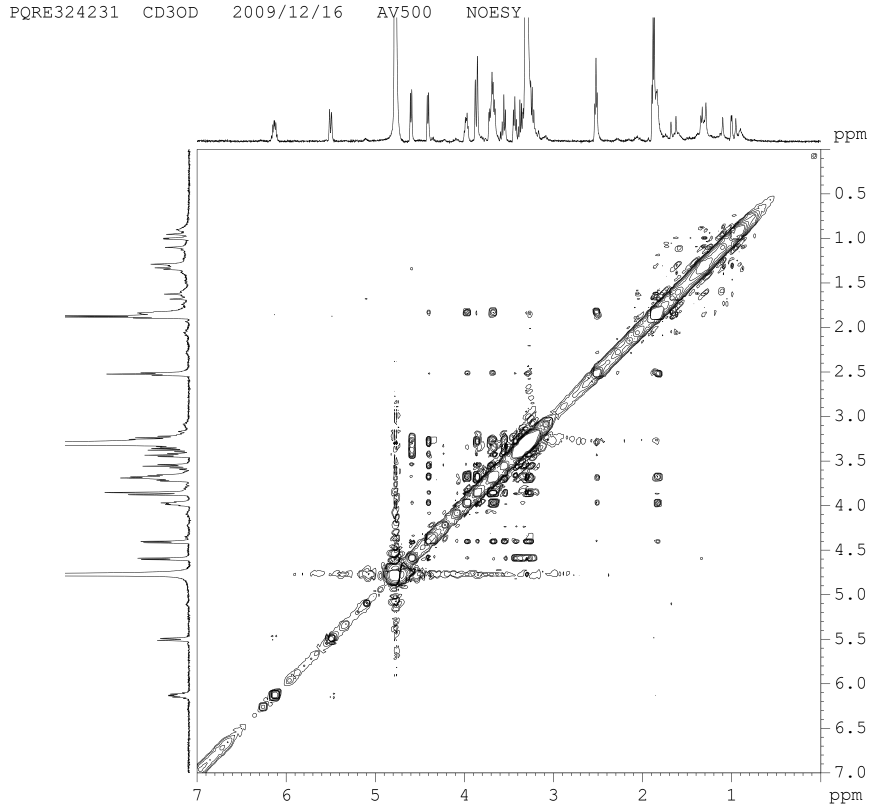

Figure S25. ESI-MS of compound **22**.

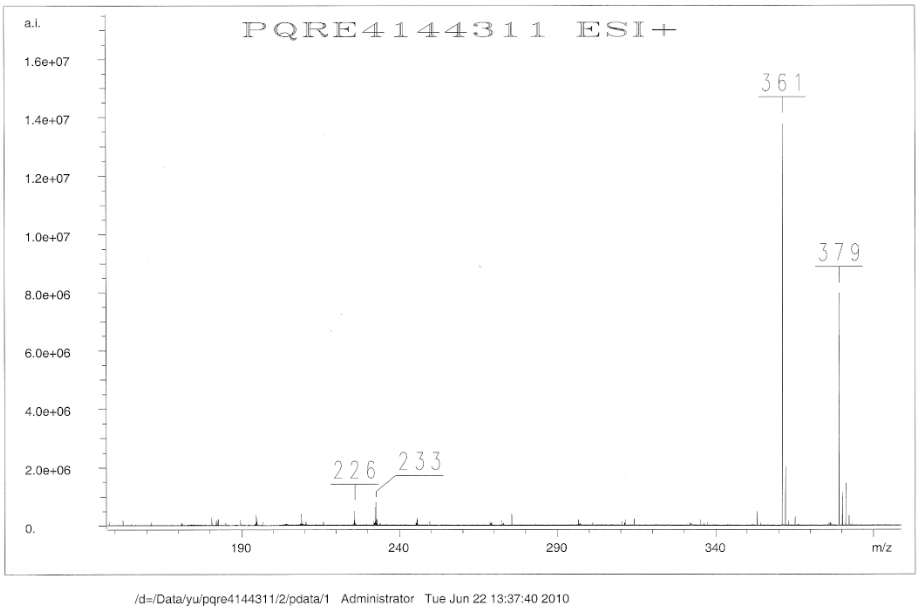

Figure S26. ^{1}H-NMR of compound **22**.

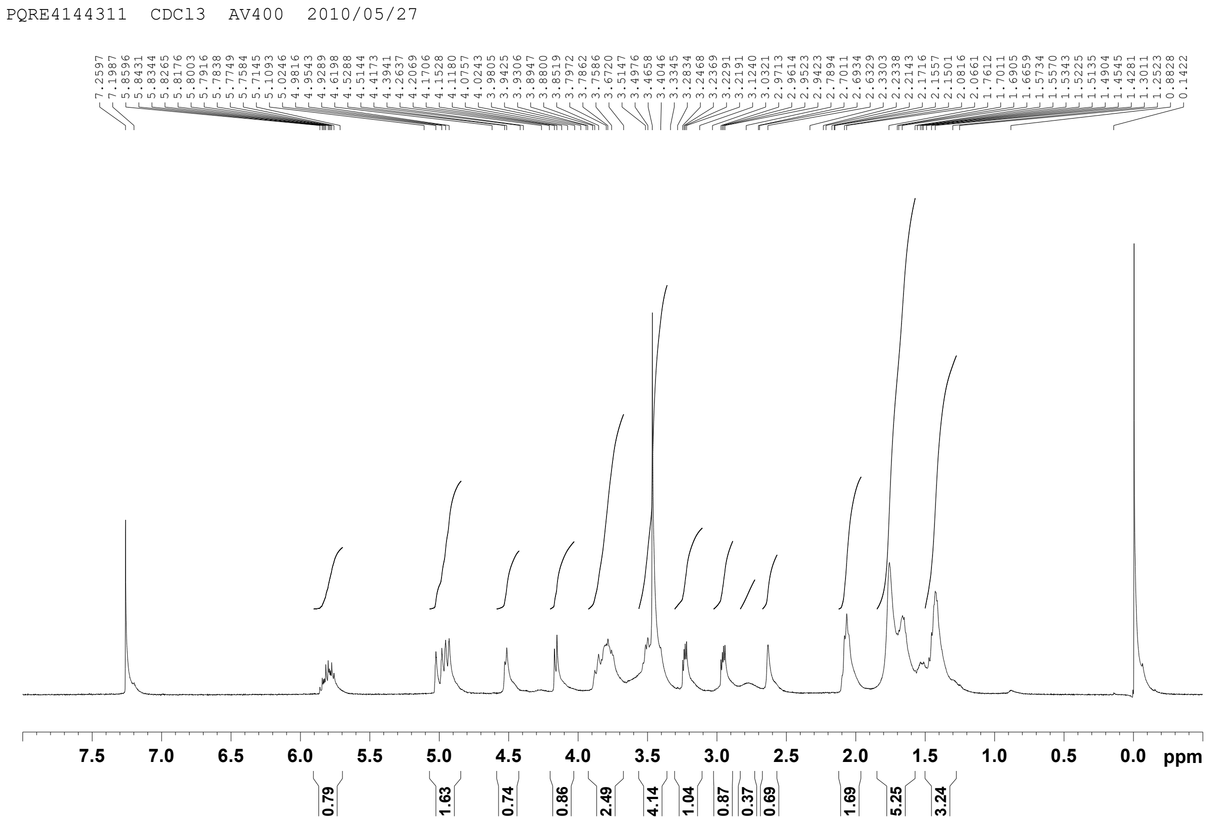

Figure S27. ^{13}C-NMR of compound **22**.

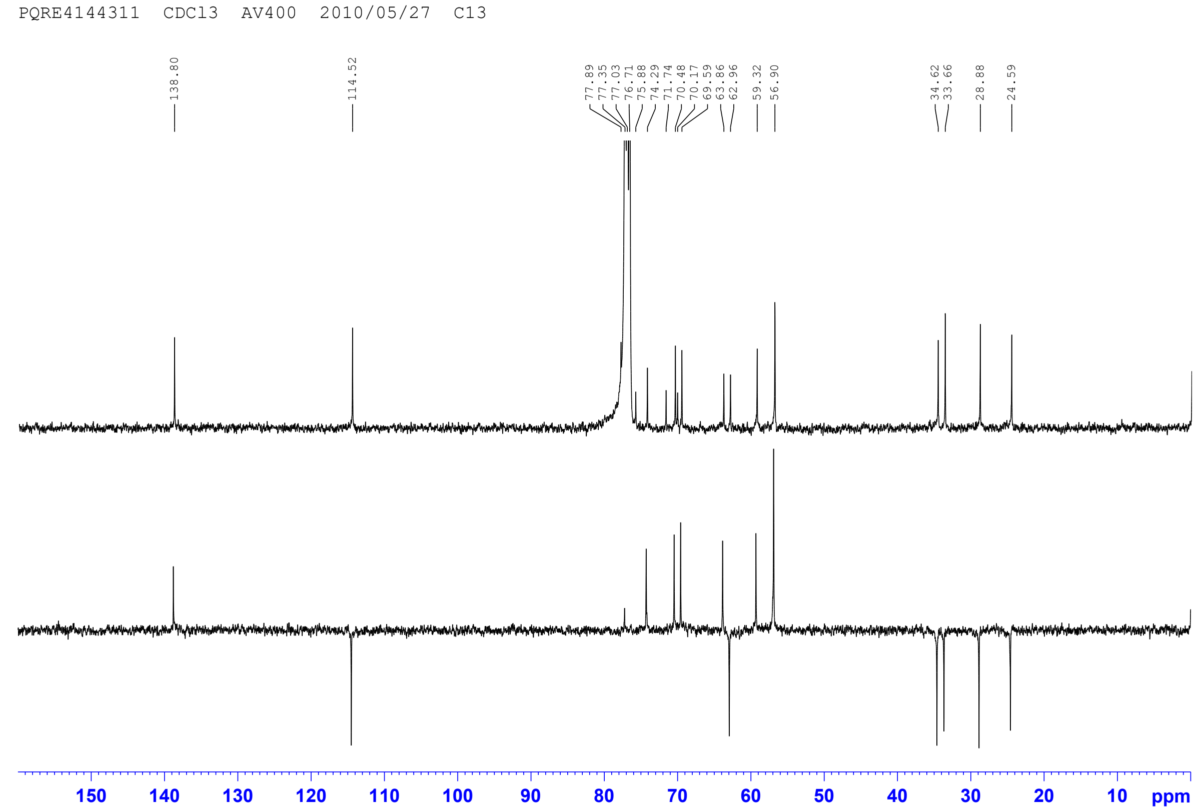

Figure S28. HMQC of compound **22**.

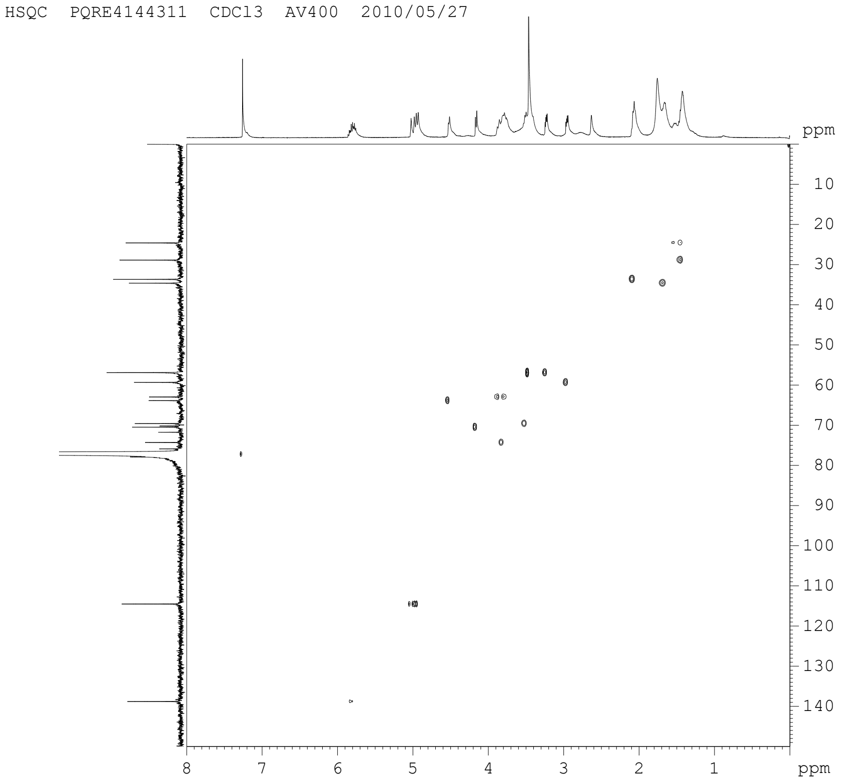

Figure S29. COSY of compound **22**.

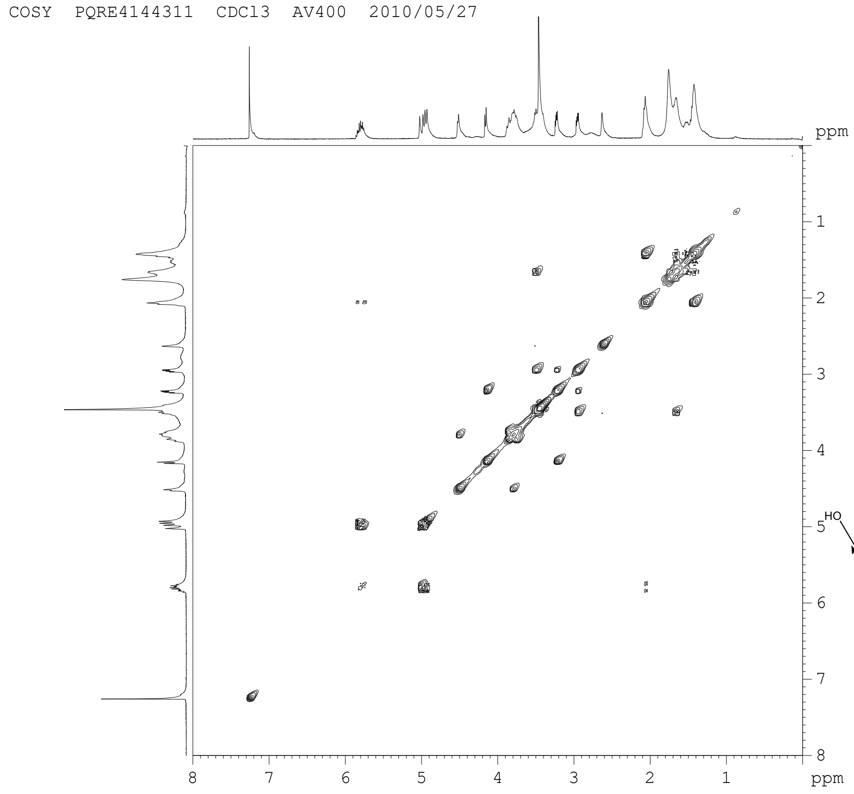

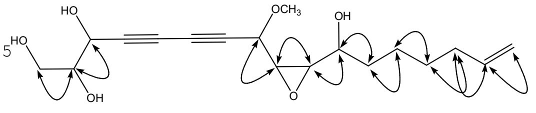

Figure S30. HMBC of compound **22**.

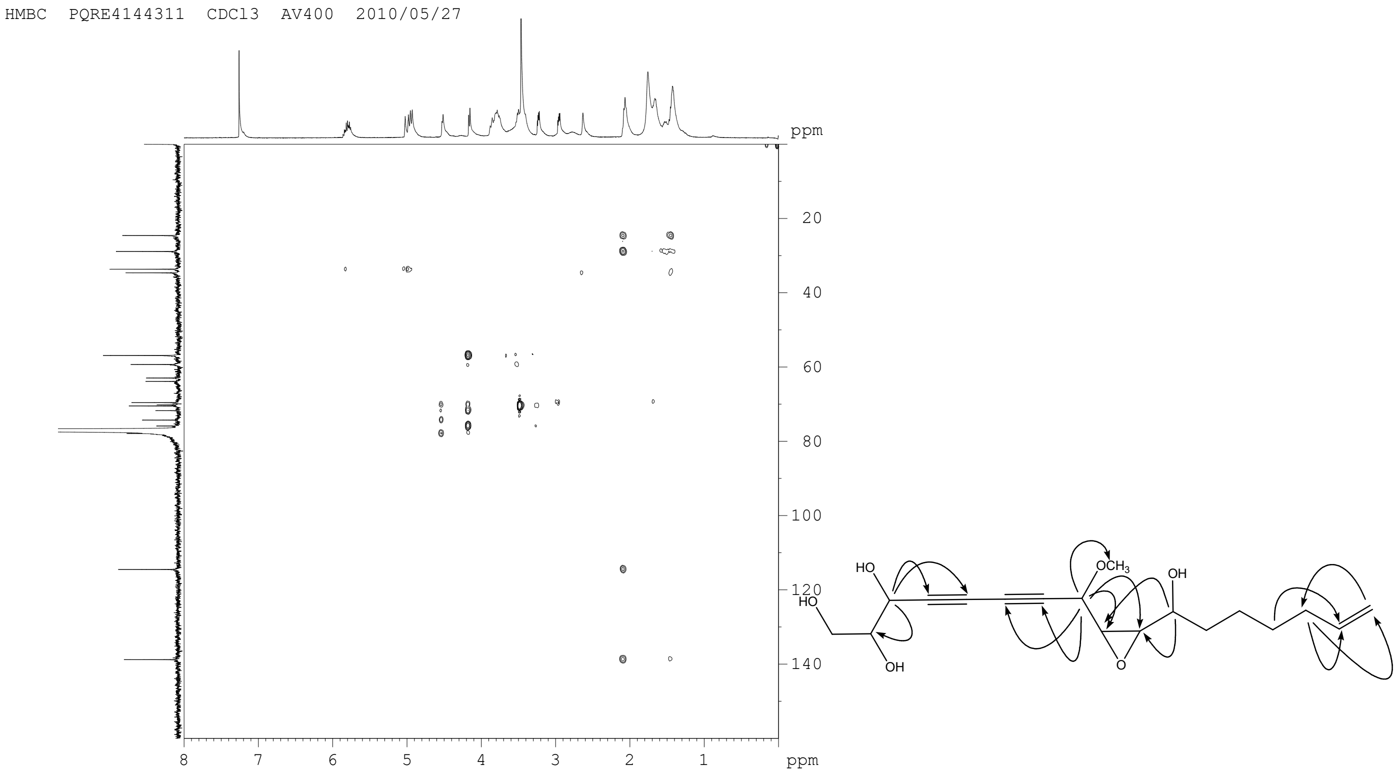

Figure S31. NOESY of compound **22**.

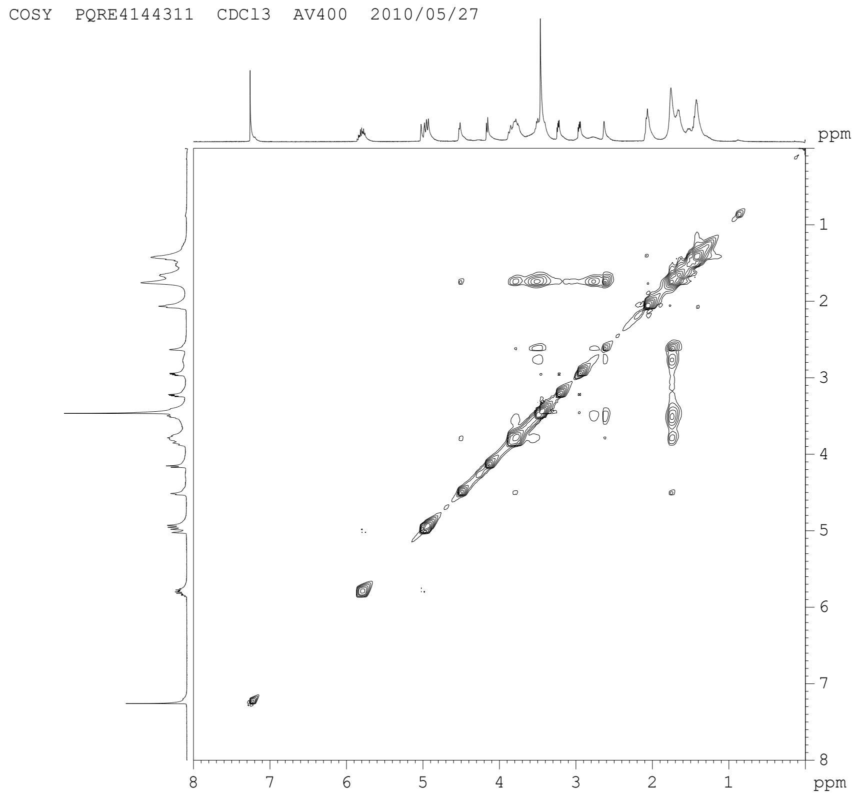

Figure S32. ^{1}H-NMR of compound **23**.

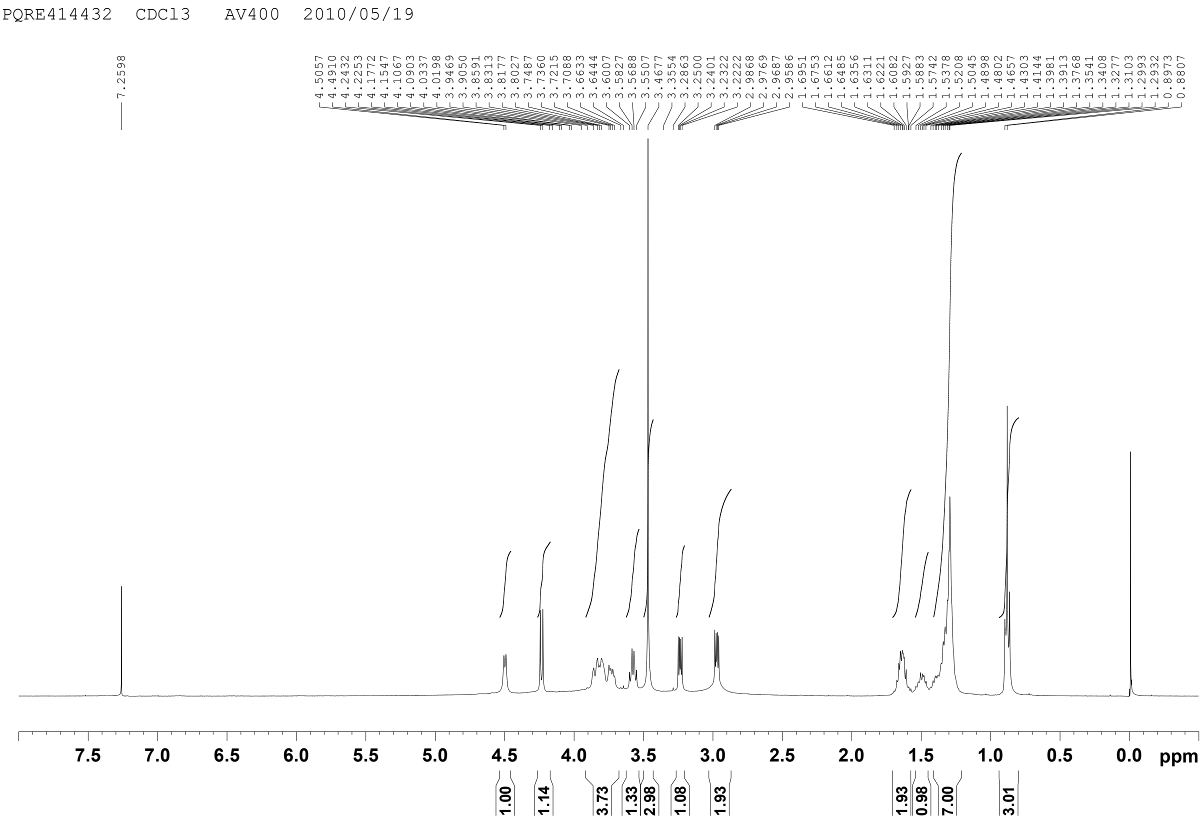

Figure S33. ^{13}C-NMR of compound **23**.

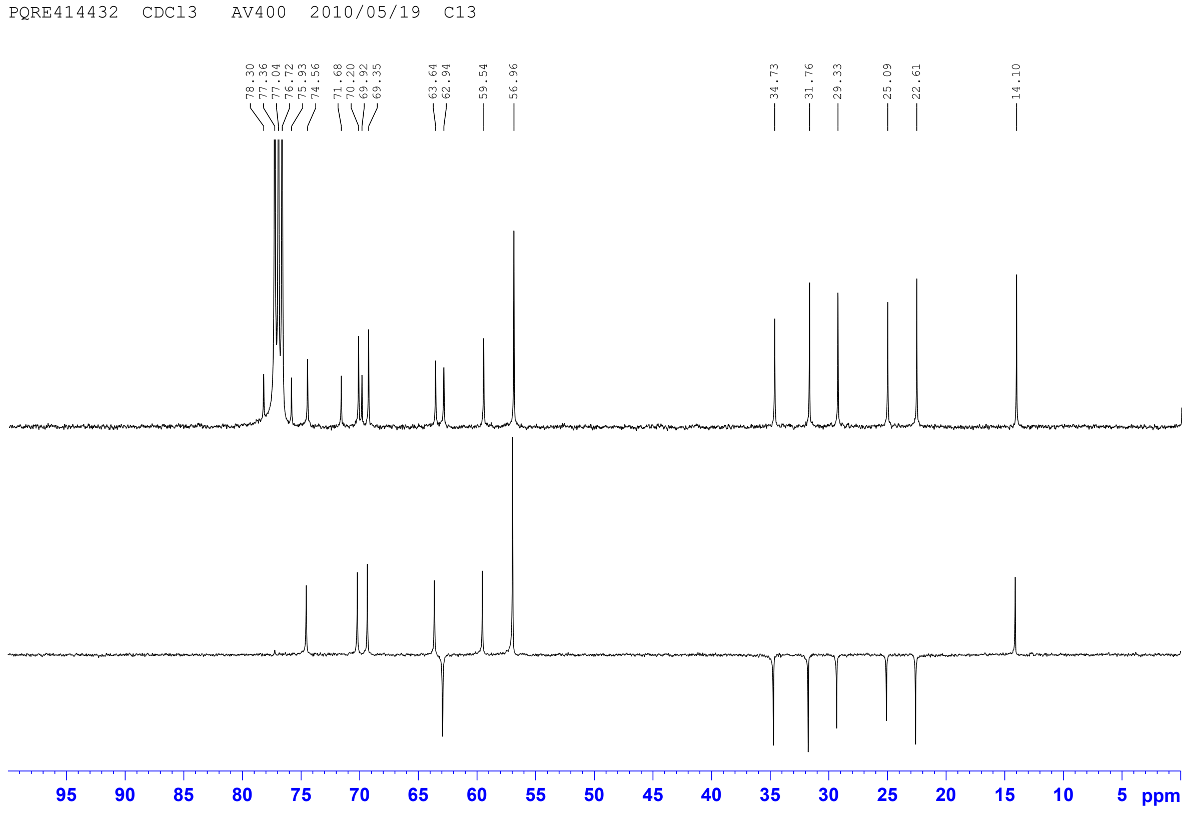

Figure S34. ESI-MS of compound **23**.

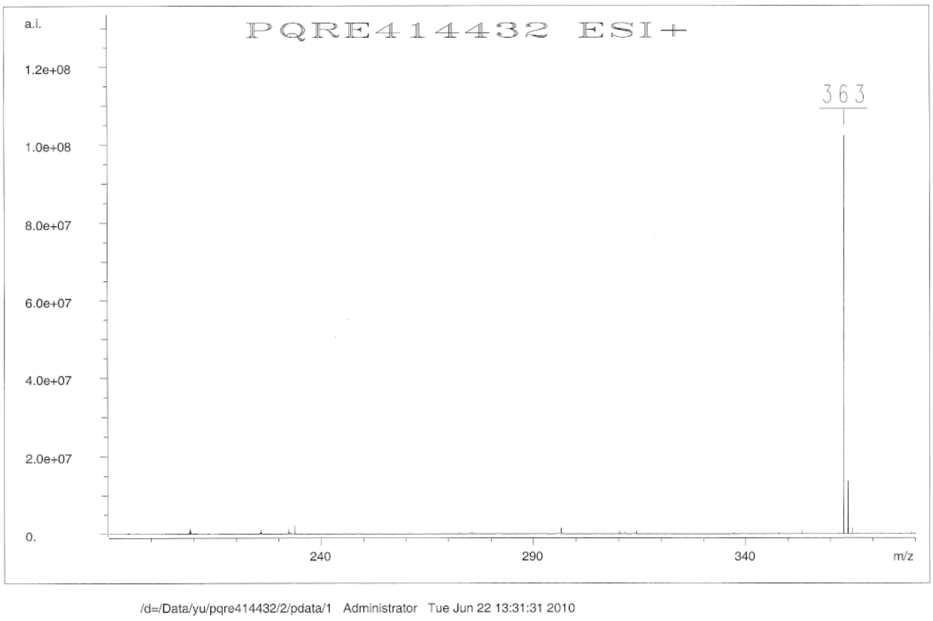

Figure S35. HMQC of compound **23**.

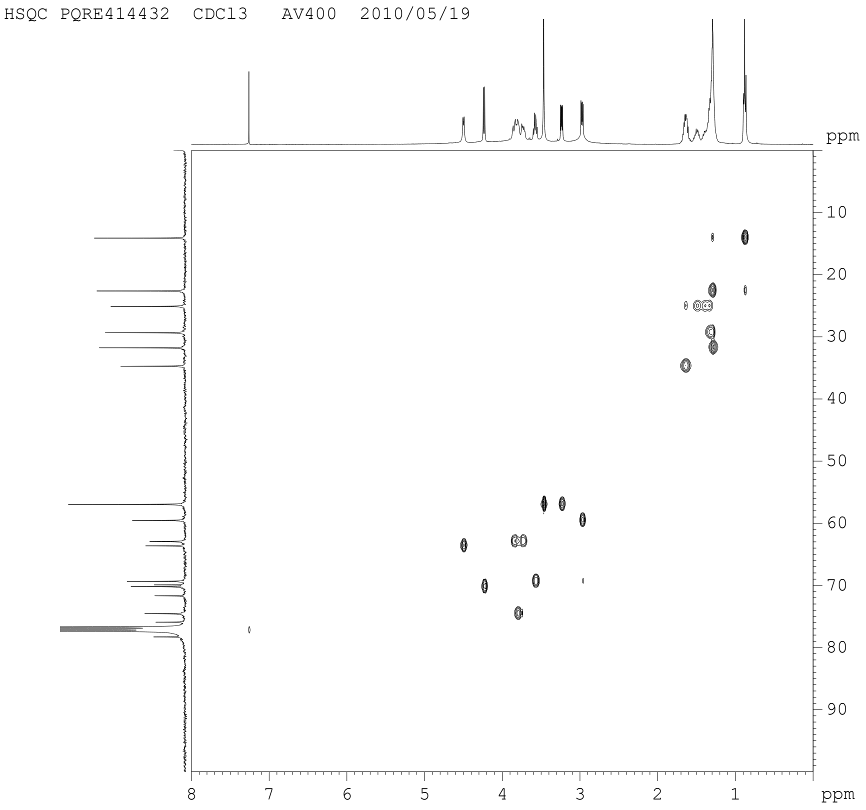

Figure S36. COSY of compound **23**.

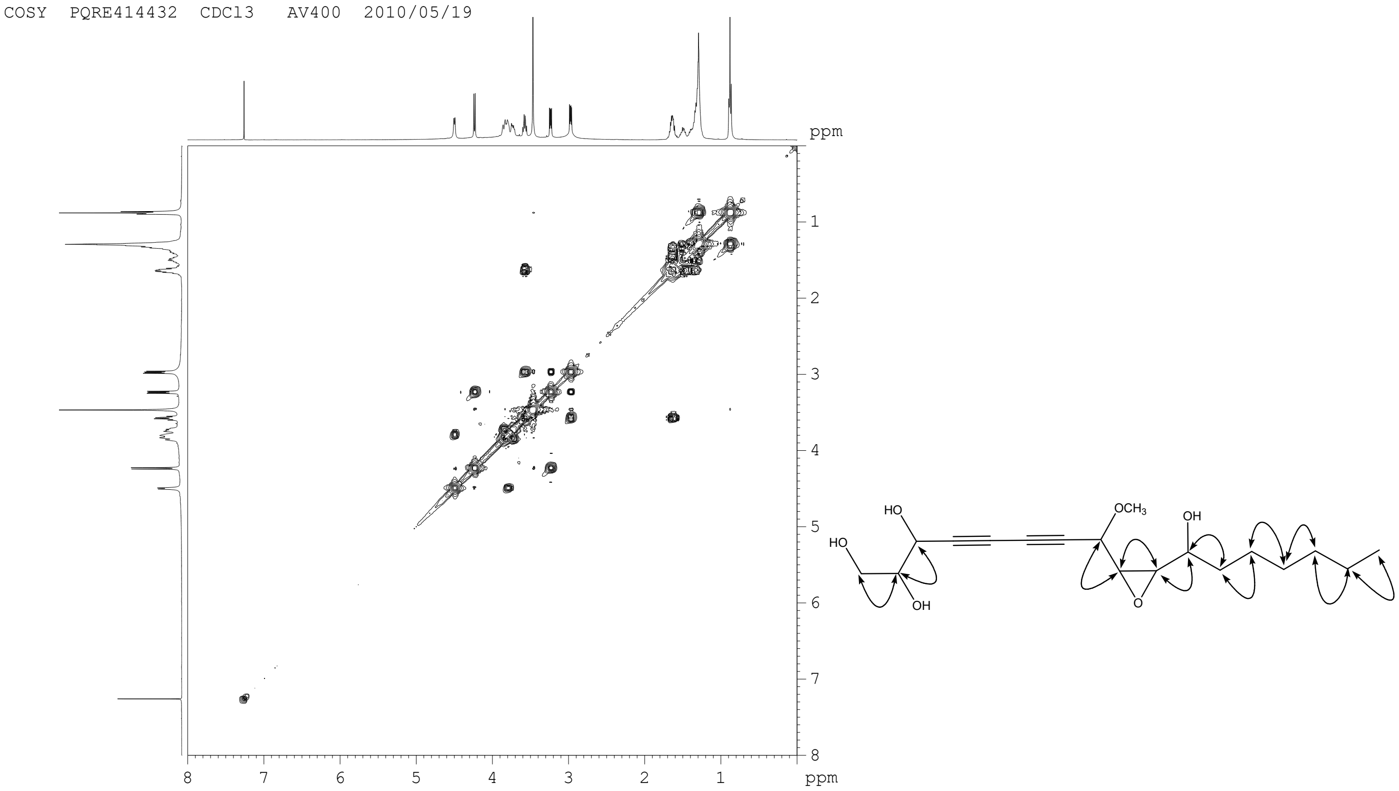

Figure S37. HMBC of compound **23**.

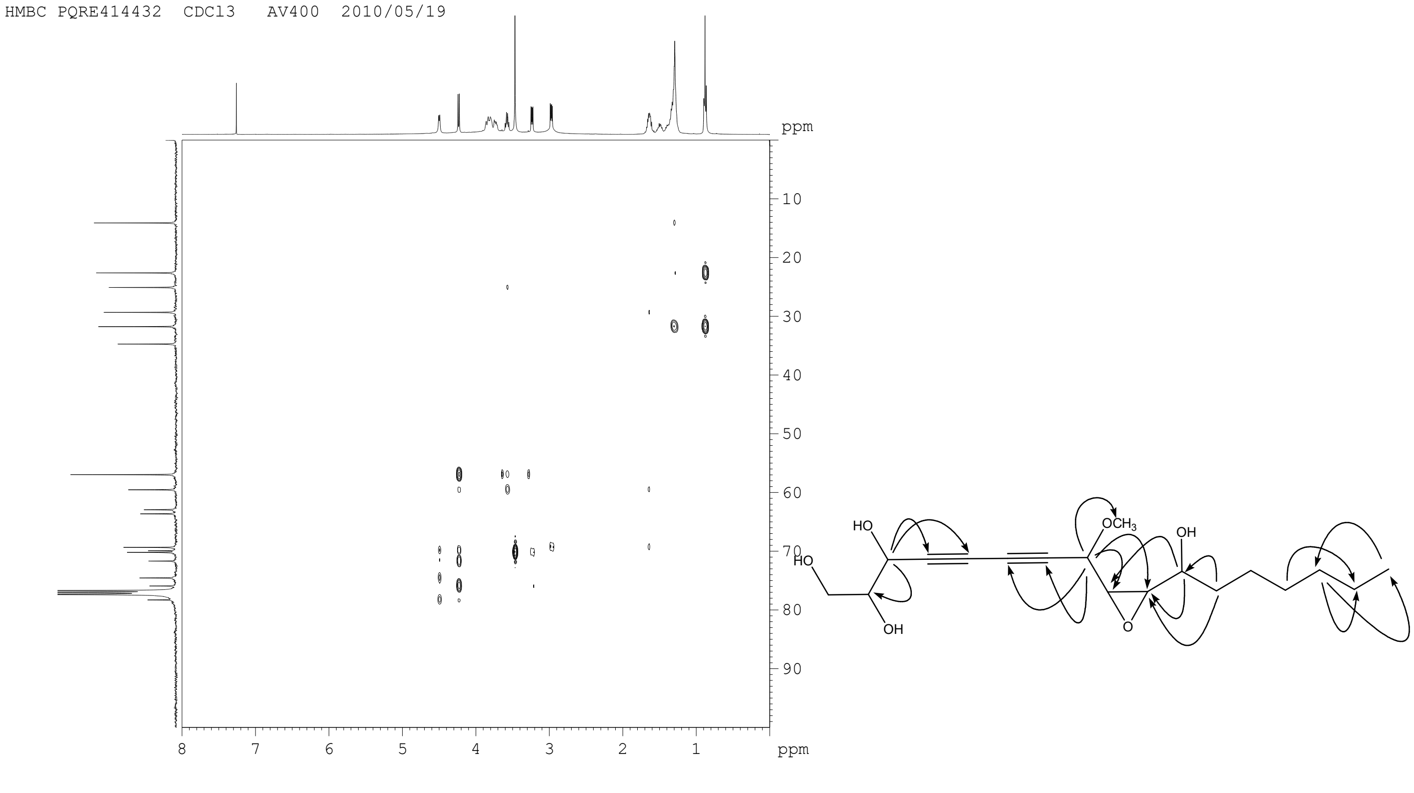

Figure S38. NOESY of compound **23**.

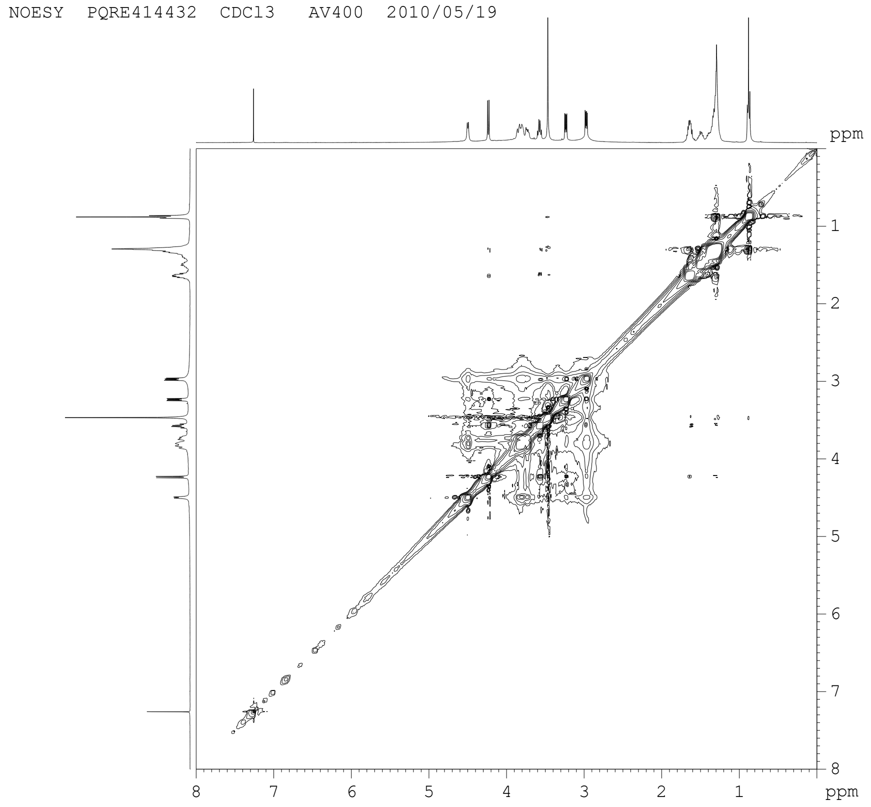

Figure S39. Basic structures of naturally purified triterpenoid saponins.

Dammarane type

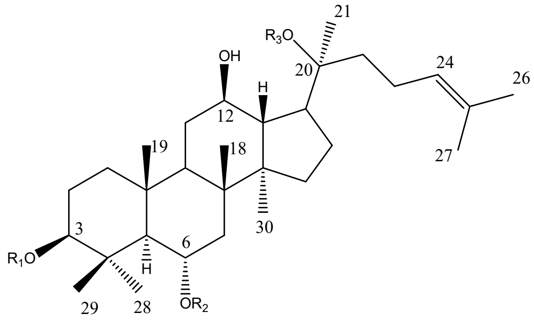

protopanaxtriol

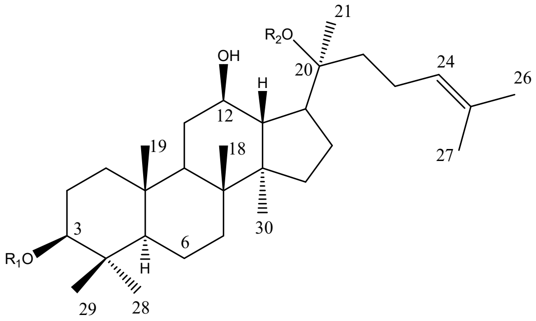

protopanaxdiol

Ocotillol type

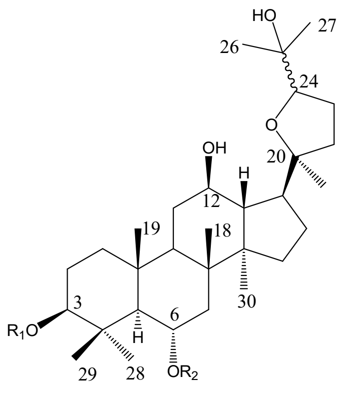

Oleanolic acid type

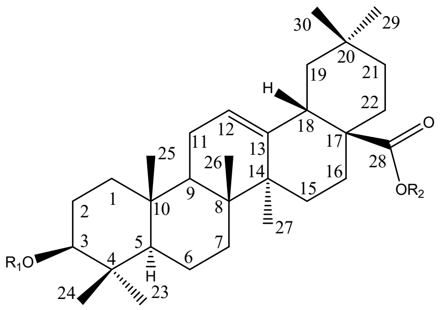

Figure S40. Biosynthetic pathway of triterpenoids.

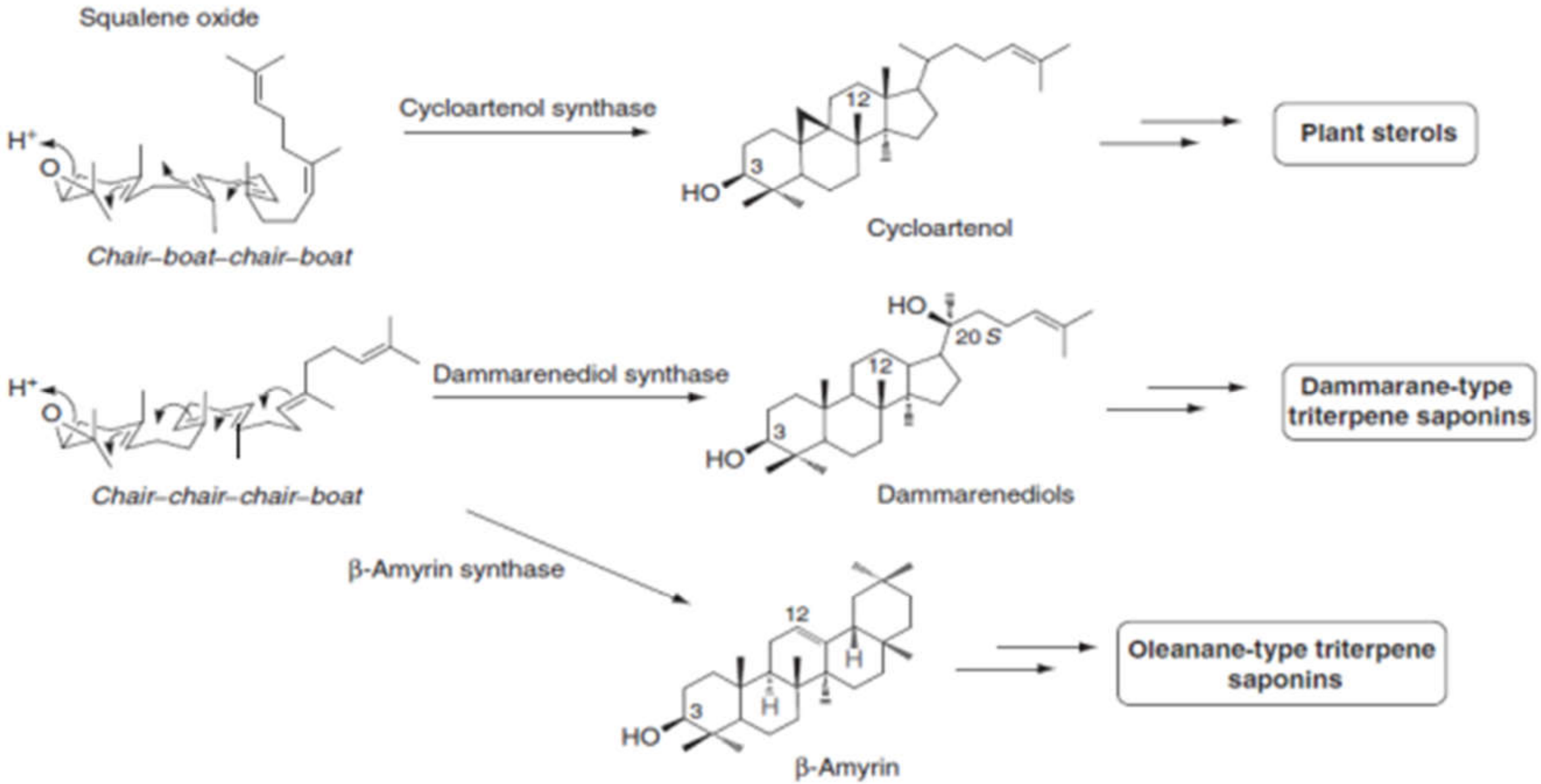

Figure S41. Biosynthetic pathway of dammarenediol-type triterpenoids.

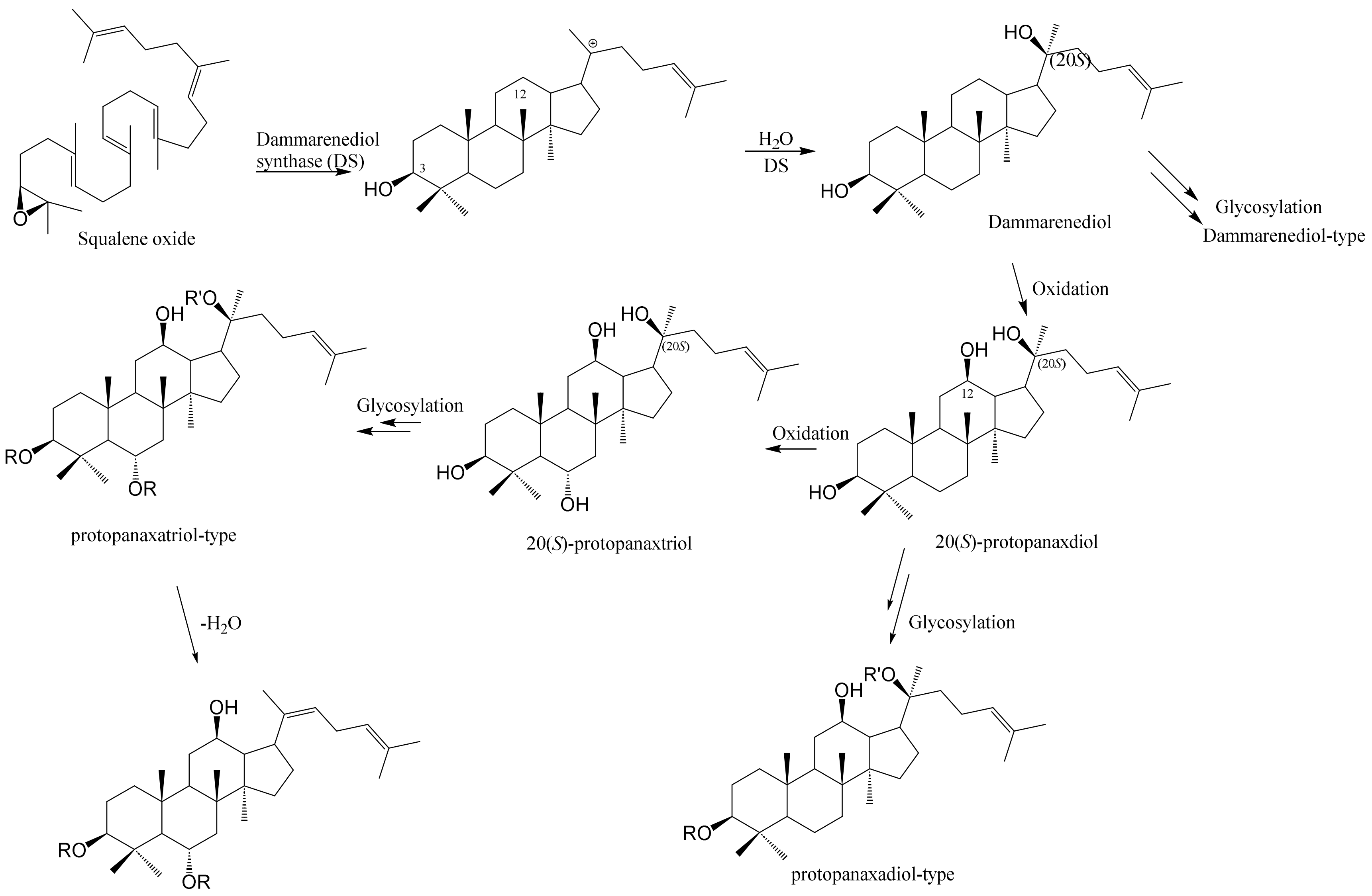

Figure S42. Biosynthetic pathway of protopanaxatriol, panaxatriol, and cotillol types triterpenoids.

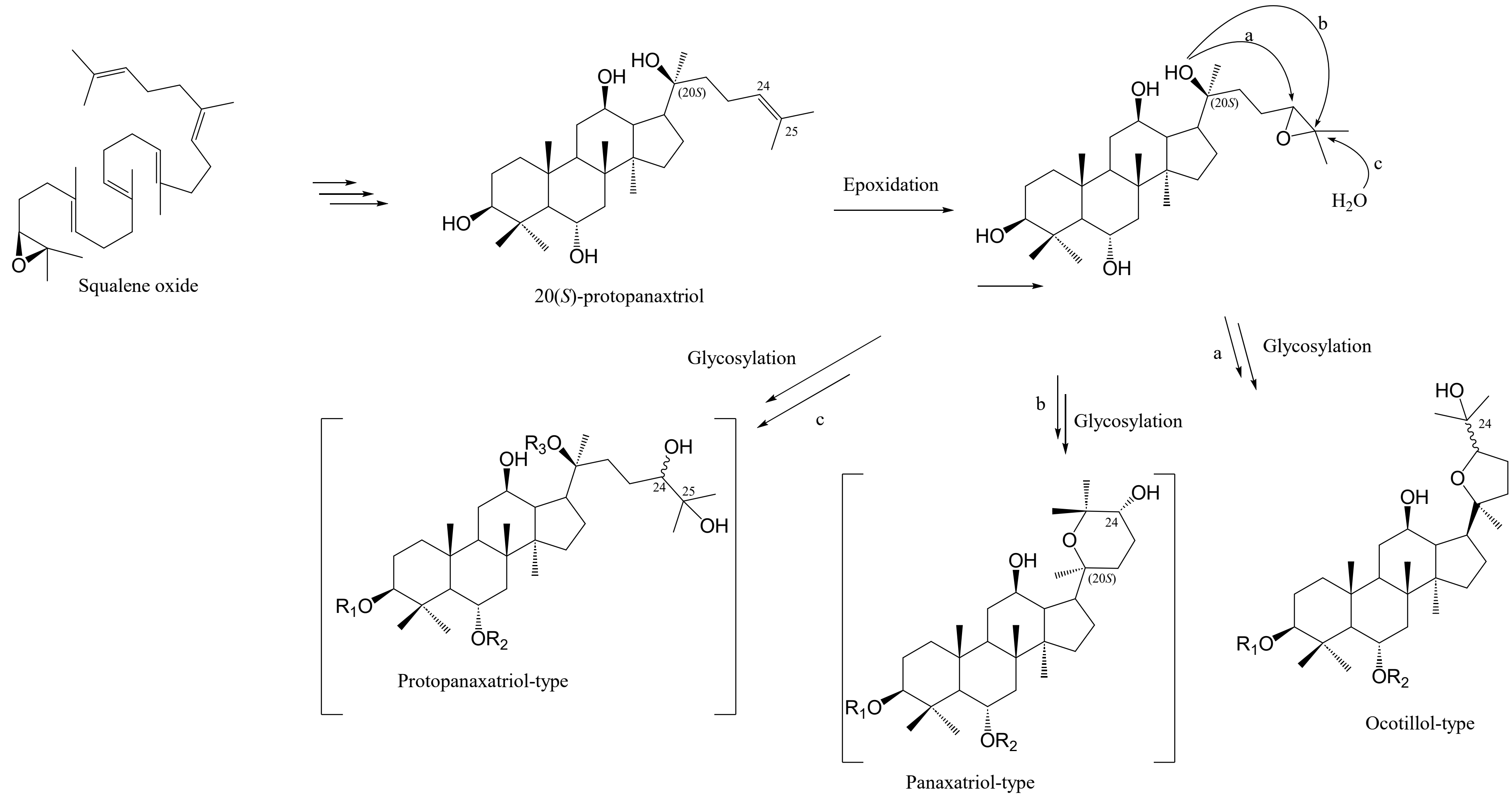

Figure S43. Biosynthetic pathway of oleanolic acid type ginsenosides.

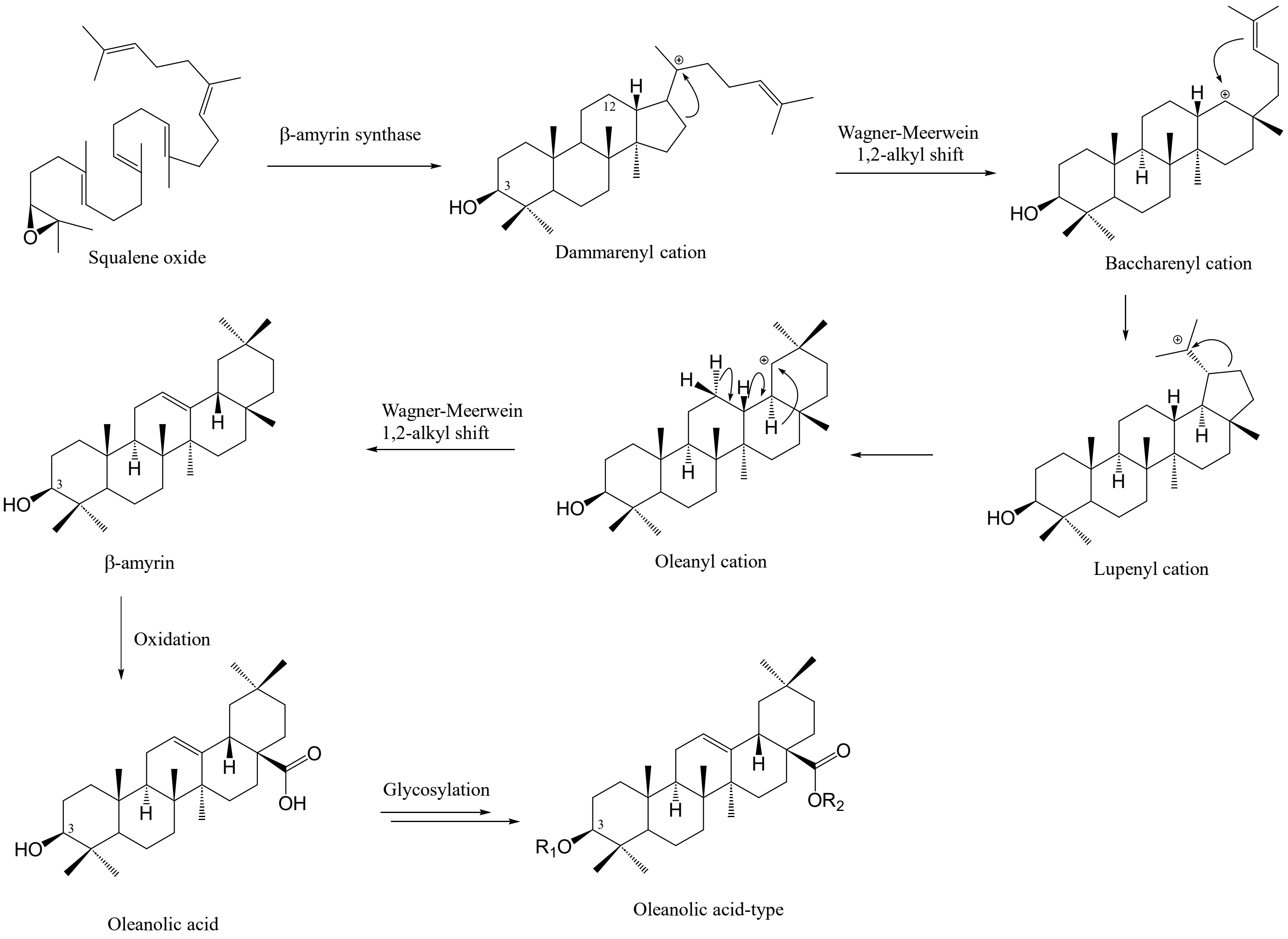

Supplement: Supplementary file 1 — ao4c04965_si_001.pdf [file ao4c04965_si_001.pdf]
